# Human-like systematic generalization through a meta-learning neural network

**DOI:** 10.1038/s41586-023-06668-3

**Published:** 2023-10-25

**Authors:** Brenden M. Lake, Marco Baroni

**Affiliations:** 1 Department of Psychology and Center for Data Science, New York University, New York, NY USA; 2 Catalan Institution for Research and Advanced Studies (ICREA), Barcelona, Spain; 3 Department of Translation and Language Sciences, Universitat Pompeu Fabra, Barcelona, Spain

**Keywords:** Human behaviour, Computer science

## Abstract

The power of human language and thought arises from systematic compositionality—the algebraic ability to understand and produce novel combinations from known components. Fodor and Pylyshyn^1^ famously argued that artificial neural networks lack this capacity and are therefore not viable models of the mind. Neural networks have advanced considerably in the years since, yet the systematicity challenge persists. Here we successfully address Fodor and Pylyshyn’s challenge by providing evidence that neural networks can achieve human-like systematicity when optimized for their compositional skills. To do so, we introduce the meta-learning for compositionality (MLC) approach for guiding training through a dynamic stream of compositional tasks. To compare humans and machines, we conducted human behavioural experiments using an instruction learning paradigm. After considering seven different models, we found that, in contrast to perfectly systematic but rigid probabilistic symbolic models, and perfectly flexible but unsystematic neural networks, only MLC achieves both the systematicity and flexibility needed for human-like generalization. MLC also advances the compositional skills of machine learning systems in several systematic generalization benchmarks. Our results show how a standard neural network architecture, optimized for its compositional skills, can mimic human systematic generalization in a head-to-head comparison.

## Abstract (web summary)

The meta-learning for compositionality approach achieves the systematicity and flexibility needed for human-like generalization.

## Main

People are adept at learning new concepts and systematically combining them with existing concepts. For example, once a child learns how to ‘skip’, they can understand how to ‘skip backwards’ or ‘skip around a cone twice’ due to their compositional skills. Fodor and Pylyshyn^1^ argued that neural networks lack this type of systematicity and are therefore not plausible cognitive models, leading to a vigorous debate that spans 35 years^2–5^. Counterarguments to Fodor and Pylyshyn^1^ have focused on two main points. The first is that human compositional skills, although important, may not be as systematic and rule-like as Fodor and Pylyshyn indicated^3,6,7^. The second is that neural networks, although limited in their most basic forms, can be more systematic when using sophisticated architectures^8–10^. In recent years, neural networks have advanced considerably and led to a number of breakthroughs, including in natural language processing. In light of these advances, we and other researchers have reformulated classic tests of systematicity and reevaluated Fodor and Pylyshyn’s arguments^1^. Notably, modern neural networks still struggle on tests of systematicity^11–18^—tests that even a minimally algebraic mind should pass^2^. As the technology marches on^19,20^, the systematicity debate continues.

**Fig. 1:**
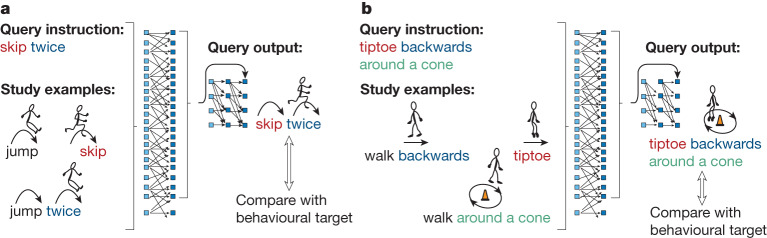
MLC for acquiring compositional skills through a dynamic stream of compositional tasks (episodes). **a**, During training, episode **a** presents a neural network with a set of study examples and a query instruction, all provided as a simultaneous input. The study examples demonstrate how to ‘jump twice’, ‘skip’ and so on with both instructions and corresponding outputs provided as words and text-based action symbols (solid arrows guiding the stick figures), respectively. The query instruction involves compositional use of a word (‘skip’) that is presented only in isolation in the study examples, and no intended output is provided. The network produces a query output that is compared (hollow arrows) with a behavioural target. **b**, Episode **b** introduces the next word (‘tiptoe’) and the network is asked to use it compositionally (‘tiptoe backwards around a cone’), and so on for many more training episodes. The colours highlight compositional reuse of words. Stick figures were adapted from art created by D. Chappard (OpenClipArt.org).

In this Article, we provide evidence that neural networks can achieve human-like systematic generalization through MLC—an optimization procedure that we introduce for encouraging systematicity through a series of few-shot compositional tasks (Fig. 1). Our implementation of MLC uses only common neural networks without added symbolic machinery, and without hand-designed internal representations or inductive biases. Instead, MLC provides a means of specifying the desired behaviour through high-level guidance and/or direct human examples; a neural network is then asked to develop the right learning skills through meta-learning^21^.

To demonstrate the abilities of MLC, we evaluated humans and machines side by side on the same tests of systematic generalization. Specifically, we used instruction-learning tasks in a pseudolanguage to examine human and machine learning of structured algebraic systems (details of the procedures are provided in the ‘Behavioural methods: few-shot learning task’ section of the Methods). We also examined behaviour in response to highly ambiguous linguistic probes, designed to characterize human inductive biases and how these biases could either facilitate or hamper systematic generalization (see the ‘Behavioural methods: open-ended task’ section of the Methods). Across these evaluations, MLC achieves (or even exceeds) human-level systematic generalization. MLC also produces human-like patterns of errors when human behaviour departs from purely algebraic reasoning, showing how neural networks are not only a capable but also a superior modelling tool for nuanced human compositional behaviour (see ‘Modelling results’). In a final set of simulations (see the ‘Machine learning benchmarks’ section of the Methods), we show how MLC improves accuracy on popular benchmarks^11,16^ for few-shot systematic generalization.

## Behavioural results

First, we measured human systematic generalization, going beyond classic work that relied primarily on thought experiments to characterize human abilities^1–3^. Our experimental paradigm asks participants to process instructions in a pseudolanguage in order to generate abstract outputs (meanings), differing from artificial grammar learning^22^, statistical learning^23^ and program learning^24^ in that explicit or implicit judgments of grammaticality are not needed. Instead, the participants generate sequences of symbols in response to sequences of words, enabling computational systems to directly model the resulting data by building on the powerful sequence-to-sequence (seq2seq) toolkit from machine learning^25,26^. All experiments were run on Amazon Mechanical Turk, and detailed procedures are described in the ‘Behavioural methods: few-shot learning task’ and ‘Behavioural methods: open-ended task’ sections of the Methods. The complete set of human and machine responses is viewable online (Data availability).

**Fig. 2:**
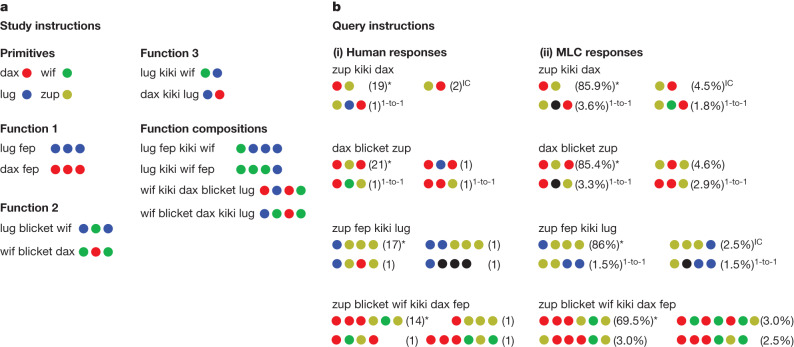
Few-shot instruction-learning task that involves responding to instructions (linguistic strings) by generating sequences of abstract outputs (coloured circles). **a**,**b**, Based on the study instructions (**a**; headings were not provided to the participants), humans and MLC executed query instructions (**b**; 4 of 10 shown). The four most frequent responses are shown, marked in parentheses with response rates (counts for people and the percentage of samples for MLC). The superscript notes indicate the algebraic answer (asterisks), a one-to-one error (1-to-1) or an iconic concatenation error (IC). The words and colours were randomized for each participant and a canonical assignment is therefore shown here. A black circle indicates a colour that was unused in the study set.

Systematic generalization was evaluated through a few-shot learning paradigm. As illustrated in Fig. 2, the participants (*n* = 25) were provided with a curriculum of 14 study instructions (input/output pairs) and asked to produce outputs for 10 query instructions (see the ‘Behavioural methods: few-shot learning task’ section of the Methods). The study instructions were consistent with an underlying interpretation grammar, which derives outputs from inputs through a set of compositional rewrite rules (see the ‘Interpretation grammars’ section of the Methods). To perform well, the participants must learn the meaning of words from just a few examples and generalize to more complex instructions. The participants were able to produce output sequences that exactly matched the algebraic standard in 80.7% of cases (indicated by an asterisk in Fig. 2b (i)). Chance performance is 2.8% for two-length output sequences if the length is known, and exponentially less for longer sequences. Notably, participants also generalized correctly in 72.5% of cases to longer output sequences than seen during training (an example is shown as the last instruction in Fig. 2b (i)), which is a type of generalization that neural networks often struggle with^11^. When deviating from this algebraic standard, the responses were still highly non-random and suggestive of strong inductive biases. Many errors involved ‘one-to-one’ translations that mapped each input word to exactly one output symbol, as if all words were primitives rather than functions (24.4% of all errors; marked with 1-to-1 in Fig. 2b (i)). Other errors involved applying a function but mixing up its arguments, often in ways that suggest an ‘iconic concatenation’ bias for maintaining the order of the input words in the order of the output symbols (23.3% of all errors involving function 3 followed this pattern; marked with IC in Fig. 2b (i)). These response patterns can be compared to biases in language acquisition more generally; indeed, forms of one-to-one^27^ and iconic concatenation^28,29^ are widely attested in natural language.

**Fig. 3:**
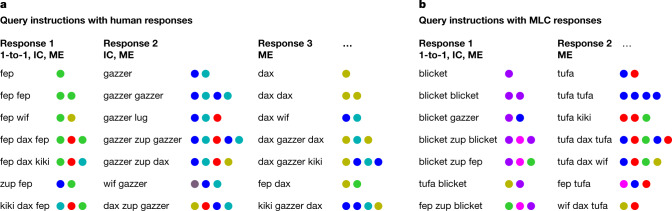
Open-ended instruction task. **a**,**b**, The participants produced responses (sequences of coloured circles) to the queries (linguistic strings) without seeing any study examples. Each column shows a different word assignment and a different response, either from a different participant (**a**) or MLC sample (**b**). The leftmost pattern (in both **a** and **b**) was the most common output for both people and MLC, translating the queries in a one-to-one (1-to-1) and left-to-right manner consistent with iconic concatenation (IC). The rightmost patterns (in both **a** and **b**) are less clearly structured but still generate a unique meaning for each instruction (mutual exclusivity (ME)).

These inductive biases were evaluated more directly through an open-ended instruction task in which the participants were not influenced by study examples and, therefore, their a priori preferences are more likely to shine through. Different human participants (*n* = 29) were asked to make plausible guesses regarding the outputs of seven unknown instructions and how they relate to one another (responding to ‘fep fep’ or ‘fep wif’ with a series of coloured circles), without seeing any input/output examples to influence their responses (see Fig. 3 for the full task and the ‘Behavioural methods: open-ended task’ section of the Methods for details). Despite the unconstrained nature of the test, people’s responses were highly structured and confirm the previous two inductive biases. People’s responses also followed a third bias related to mutual exclusivity that encourages assigning unique meanings to unique words^27^. Reflecting the strong influence of the biases, the majority of participants (17 out of 29; 58.6%) responded with a pattern analogous to that in Fig. 3a,b (left), which is perfectly consistent with all three inductive biases. Across all responses, 18 out of 29 participants followed one-to-one (62.1%), 23 out of 29 (79.3%) followed iconic concatenation and all but two followed mutual exclusivity in producing a unique response to each instruction (27 out of 29; 93.1%).

## Modelling results

We next evaluated MLC on its ability to produce human-level systematic generalization and human-like patterns of error on these challenging generalization tasks. A successful model must learn and use words in systematic ways from just a few examples, and prefer hypotheses that capture structured input/output relationships. MLC aims to guide a neural network to parameter values that, when faced with an unknown task, support exactly these kinds of generalizations and overcome previous limitations for systematicity. Importantly, this approach seeks to model adult compositional skills but not the process by which adults acquire those skills, which is an issue that is considered further in the general discussion. MLC source code and pretrained models are available online (Code availability).

**Fig. 4:**
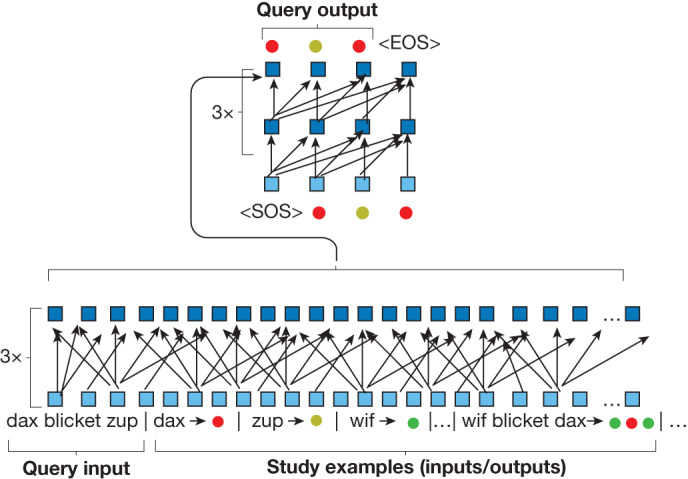
MLC architecture. A standard transformer encoder (bottom) processes the query input along with a set of study examples (input/output pairs; examples are delimited by a vertical line (∣) token). The standard decoder (top) receives the encoder’s messages and produces an output sequence in response. After optimization on episodes generated from various grammars, the transformer performs novel tasks using frozen weights. Each box is an embedding (vector); input embeddings are light blue (latent are dark).

As shown in Fig. 4 and detailed in the ‘Architecture and optimizer’ section of the Methods, MLC uses the standard transformer architecture^26^ for memory-based meta-learning. MLC optimizes the transformer for responding to a novel instruction (query input) given a set of input/output pairs (study examples; also known as support examples^21^), all of which are concatenated and passed together as the input. This amounts to meta-learning because optimization occurs over dynamically changing episodes (each with new study and query examples) rather than a static dataset; specifically, each episode constitutes a different seq2seq task^30,31^ defined through a randomly generated latent grammar for interpreting inputs as outputs (see the ‘Meta-training procedures for MLC and MLC variants’ section of the Methods). To succeed, the transformer must find parameter values that are capable of extracting meanings from the study words and composing them to answer queries, relying on meta-learning but also innovations in the transformer architecture that were not envisioned in Fodor and Pylyshyn’s arguments^1^ (for example, variable length input, parameter sharing and self-attention). On test episodes, the model weights are frozen and no task-specific parameters are provided^32^. Finally, given the end goal of modelling human responses (including errors), we stochastically pair each query with either the algebraic output sequence (generated through the episode’s grammar) or a heuristic output sequence (sampled through one-to-one translations or misapplied rules), at approximately the same ratios as observed empirically (see the ‘Meta-training procedures for MLC and MLC variants’ section of the Methods).

**Extended Data Fig. 1:**
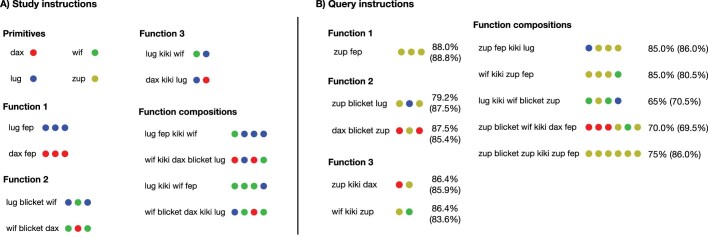
Few-shot instruction learning task with full set of queries. Based on the study instructions (A; headings were not provided to participants), humans and the MLC model executed 10 query instructions by generating coloured circles from a fixed inventory (B; headings were not provided to participants). The percent of participants who produced each sequence exactly as prescribed algebraically is shown. Similarly, the percent of samples from MLC that match the prescribed sequence is shown in parentheses, which correlates with the human values (Pearson’s *r* = 0.788, *p* = 0.031 via permutation test, two-tailed, *n* = 10 items). The words and colours were randomized for each participant.

MLC is capable of optimizing models for highly systematic behaviour. The most systematic run produced a transformer that was perfectly systematic (100% exact match accuracy) when choosing the best responses on the same few-shot instruction-learning task given to people (Fig. 2; see the ‘Evaluation procedures’ section of the Methods for details and Supplementary Information 1 for model variability across 10 runs) and additionally capable of inferring novel rules that did not participate in meta-learning (Supplementary Information 1). An informal analysis of this run further shows that MLC is also capable of more subtle and bias-driven behaviours; when sampling from the distribution of model outputs (Fig. 2b), the transformer produced systematic outputs at an average rate (82.4%) close to human performance (80.7%), and appropriately handled longer output sequences at a rate (77.8%) near human levels (72.5%). Moreover, like people, the MLC transformer made errors reflecting one-to-one translations (56.3% of errors; 24.4% for people) and iconic concatenations (13.8% of errors involving function 3; 23.3% for people). MLC can also predict which instructions are easier or harder for people on average (Pearson’s *r* = 0.788, *P* = 0.031, two-tailed permutation test, *n* = 10 items; item-level performance is shown in Extended Data Fig. 1). Formally, in Table 1 (few-shot learning), we compare models through the log-likelihood of all the human responses (Fig. 2b (i)) given the model predictions^33^. In the rest of this paragraph, when we say that one model outperforms another, there is a difference of 8 natural log points or greater. The MLC transformer (Table 1; MLC) outperforms more rigidly systematic models at predicting human behaviour. This includes a probabilistic symbolic model that assumes that people infer the gold grammar but make occasional arbitrary lapses (symbolic (oracle); details of all of the symbolic and basic seq2seq models are provided in the ‘Alternative neural and symbolic models’ section of the Methods) and a transformer optimized on the same training episodes as MLC although with strictly algebraic (rather than also bias-based) output responses (MLC (algebraic only); details of all of the MLC variants are provided in the ‘Meta-training procedures for MLC and MLC variants’ section of the Methods). MLC also outperforms a basic seq2seq transformer fit to the patterns in Fig. 2 without meta-learning and an MLC model optimized for copying rather than systematic generalization (MLC (copy only); during training, the query examples always match one of the study examples). The MLC transformer performs comparably to a probabilistic symbolic model that assumes that people infer the gold grammar but respond stochastically with lapses based on the human inductive biases (symbolic (oracle/biases)). Indeed, MLC was similarly optimized to (implicitly) infer systematic rules and respond with the same biased-based patterns, and it is therefore natural that the two models would perform similarly. The top-performing MLC (joint) was jointly optimized on both the few-shot learning task and the open-ended human responses, as described in the next paragraph.

**Table 1:** log-likelihood of human behaviour as predicted by models

Model	Few-shot learning	Open-ended
Baseline	−1,926.5	−1,547.0
Symbolic (oracle)	−538.8	–
Symbolic (oracle/biases)	−357.2	−1,008.5
Basic seq2seq	−1,264.7	–
MLC (copy only)	−1,586.4	−1,341.4
MLC (algebraic only)	−496.9	−1,218.3
MLC (joint)	**−349.2**	**−635.7**
MLC	−358.1	−693.1

All of the models have fit lapse rates (see the ‘Alternative neural and symbolic models’ section of the Methods). The baseline model samples symbols uniformly. For few-shot learning, the most systematic run was analysed (based on likelihood of gold algebraic sequences). The best scores are indicated in bold.

Although human few-shot learning behaviour can be well characterized by either MLC or a probabilistic symbolic model, a test of more open-ended behaviour emphasizes MLC’s relative strengths. The same transformer architecture was optimized on open-ended participant behaviour and then asked to fill in outputs for the seven instructions one by one (Fig. 3; see the ‘Evaluation procedures’ section of the Methods). The MLC transformer responded exactly like the modal human participant in 65.0% of samples (Fig. 3b (left)), perfectly instantiating the three key inductive biases. An informal analysis further revealed that MLC captured more nuanced patterns of response that only partially use the inductive biases (Fig. 3b (right)). Across all model samples, 66.0% followed one-to-one (62.1% for people), 85.0% followed iconic concatenation (79.3% for people) and the vast majority (99.0%) chose a unique response for each unique command (93.1% for people). Model predictions were also evaluated through fivefold cross-validation^33^: MLC and other models were optimized on responses for either 23 or 24 participants (depending on the cross-validation split) and then predicted responses for held-out participants. Performance was scored by log-likelihood and is summarized in Table 1 (open-ended) (summed over five cross-validation splits, averaged over three runs). In the rest of this paragraph, when we say that one model outperforms another, there is a difference of 57 natural log points or greater. MLC outperforms all alternatives, including the same highly algebraic MLC model as described in the previous experiment (MLC (algebraic only)) and a probabilistic symbolic model that uses the three inductive biases to generate responses but, in contrast to MLC, is not capable of optimizing for other patterns in the human behaviour (Table 1; symbolic (oracle/biases)). Importantly, a single transformer can be optimized for both the few-shot learning and open-ended instruction tasks (MLC (joint)); in fact, this is the strongest overall model across experiments for predicting human behaviour (additional analysis is shown in Extended Data Fig. 5 and Supplementary Information 1).

## Machine learning benchmarks

Beyond predicting human behaviour, MLC can achieve error rates of less than 1% on machine learning benchmarks for systematic generalization. Note that here the examples used for optimization were generated by the benchmark designers through algebraic rules, and there is therefore no direct imitation of human behavioural data. We experiment with two popular benchmarks, SCAN^11^ and COGS^16^, focusing on their systematic lexical generalization tasks that probe the handling of new words and word combinations (as opposed to new sentence structures). MLC still used only standard transformer components but, to handle longer sequences, added modularity in how the study examples were processed, as described in the ‘Machine learning benchmarks’ section of the Methods. SCAN involves translating instructions (such as ‘walk twice’) into sequences of actions (‘WALK WALK’). In the ‘add jump’ split, the training set has just one example of how to ‘jump’ (mapping to ‘JUMP’) and the test set probes compositional uses of this verb (for example, ‘jump around right twice and walk thrice’), paralleling our human learning task (‘zup’ is the analogue of ‘jump’ in Fig. 2). COGS involves translating sentences (for example, ‘A balloon was drawn by Emma’) into logical forms that express their meanings (balloon(*x*_1_) ∨ draw.theme(*x*_3_, *x*_1_) ∨ draw.agent(*x*_3_, Emma)). COGS evaluates 21 different types of systematic generalization, with a majority examining one-shot learning of nouns and verbs. To encourage few-shot inference and composition of meaning, we rely on surface-level word-type permutations for both benchmarks, a simple variant of meta-learning that uses minimal structural knowledge, described in the ‘Machine learning benchmarks’ section of the Methods. These permutations induce changes in word meaning without expanding the benchmark’s vocabulary, to approximate the more naturalistic, continual introduction of new words (Fig. 1).

The benchmark error rates are summarized in Table 2. On SCAN, MLC solves three systematic generalization splits with an error rate of 0.22% or lower (99.78% accuracy or above), including the already mentioned ‘add jump’ split and ‘around right’ and ‘opposite right’, which examine novel combinations of known words. On COGS, MLC achieves an error rate of 0.87% across the 18 types of lexical generalization. Without the benefit of meta-learning, basic seq2seq has error rates at least seven times as high across the benchmarks, despite using the same transformer architecture. However surface-level permutations were not enough for MLC to solve the structural generalization tasks in the benchmarks. MLC fails to handle longer output sequences (SCAN length split) as well as novel and more complex sentence structures (three types in COGS), with error rates at 100%. Such tasks require handling ‘productivity’ (page 33 of ref. ^1^), in ways that are largely distinct from systematicity. However, MLC did handle novel sentence structures in our few-shot instruction-learning task (77.8% correct on queries with both longer input and output sequences than seen during study; Fig. 2), suggesting that the right meta-training procedure can promote productivity—a challenge we leave to future work.

**Table 2:** Error rates for systematic lexical generalization on machine learning benchmarks

	Within-distribution generalization		Systematic lexical generalization			
Model	SCAN Simple	COGS Simple	SCAN Add jump	SCAN Around right	SCAN Opp. right	COGS Lexical
Basic seq2seq	**0.00%**	**0.12%**	99.27%	51.13%	100.00%	6.08%
MLC	0.02%	0.34%	**0.22%**	**0.04%**	**0.06%**	**0.87%**

Values are exact-match error rates averaged over five runs (0.00% is perfect and 100.00% is worst case). Simple splits evaluate within-distribution generalization. All other splits evaluate systematic lexical generalization. The best scores are indicated in bold font. Opp, opposite.

## Discussion

Over 35 years ago, when Fodor and Pylyshyn raised the issue of systematicity in neural networks^1^, today’s models^19^ and their language skills were probably unimaginable. As a credit to Fodor and Pylyshyn’s prescience, the systematicity debate has endured. Systematicity continues to challenge models^11–18^ and motivates new frameworks^34–41^. Preliminary experiments reported in Supplementary Information 3 suggest that systematicity is still a challenge, or at the very least an open question, even for recent large language models such as GPT-4. To resolve the debate, and to understand whether neural networks can capture human-like compositional skills, we must compare humans and machines side-by-side, as in this Article and other recent work^7,42,43^. In our experiments, we found that the most common human responses were algebraic and systematic in exactly the ways that Fodor and Pylyshyn^1^ discuss. However, people also relied on inductive biases that sometimes support the algebraic solution and sometimes deviate from it; indeed, people are not purely algebraic machines^3,6,7^. We showed how MLC enables a standard neural network optimized for its compositional skills to mimic or exceed human systematic generalization in a side-by-side comparison. MLC shows much stronger systematicity than neural networks trained in standard ways, and shows more nuanced behaviour than pristine symbolic models. MLC also allows neural networks to tackle other existing challenges, including making systematic use of isolated primitives^11,16^ and using mutual exclusivity to infer meanings^44^.

Our use of MLC for behavioural modelling relates to other approaches for reverse engineering human inductive biases. Bayesian approaches enable a modeller to evaluate different representational forms and parameter settings for capturing human behaviour, as specified through the model’s prior^45^. These priors can also be tuned with behavioural data through hierarchical Bayesian modelling^46^, although the resulting set-up can be restrictive. MLC shows how meta-learning can be used like hierarchical Bayesian models for reverse-engineering inductive biases (see ref. ^47^ for a formal connection), although with the aid of neural networks for greater expressive power. Our research adds to a growing literature, reviewed previously^48^, on using meta-learning for understanding human^49–51^ or human-like behaviour^52–54^. In our experiments, only MLC closely reproduced human behaviour with respect to both systematicity and biases, with the MLC (joint) model best navigating the trade-off between these two blueprints of human linguistic behaviour. Furthermore, MLC derives its abilities through meta-learning, where both systematic generalization and the human biases are not inherent properties of the neural network architecture but, instead, are induced from data.

Despite its successes, MLC does not solve every challenge raised in Fodor and Pylyshyn^1^. MLC does not automatically handle unpractised forms of generalization or concepts outside the meta-learning distribution, reducing the scope of entirely novel structures it can correctly process (compare the encouraging results on learning novel rules reported in Supplementary Information 1, with its failure on the SCAN and COGS productivity splits). Moreover, MLC is failing to generalize to nuances in inductive biases that it was not optimized for, as we explore further through an additional behavioural and modelling experiment in Supplementary Information 2. In the language of machine learning, we conclude that the meta-learning strategy succeeds when generalization makes a new episode in-distribution with respect to the training episodes, even when the specific test items are out-of-distribution with respect to the study examples in the episode. However, meta-learning alone will not allow a standard network to generalize to episodes that are in turn out-of-distribution with respect to the ones presented during meta-learning. The current architecture also lacks a mechanism for emitting new symbols^2^, although new symbols introduced through the study examples could be emitted through an additional pointer mechanism^55^. Last, MLC is untested on the full complexity of natural language and on other modalities; therefore, whether it can achieve human-like systematicity, in all respects and from realistic training experience, remains to be determined. Nevertheless, our use of standard transformers will aid MLC in tackling a wider range of problems at scale. For example, a large language model could receive specialized meta-training^56^, optimizing its compositional skills by alternating between standard training (next word prediction) and MLC meta-training that continually introduces novel words and explicitly improve systematicity (Fig. 1). For vision problems, an image classifier or generator could similarly receive specialized meta-training (through current prompt-based procedures^57^) to learn how to systematically combine object features or multiple objects with relations.

Our study raises natural developmental questions. The specific procedure of optimizing over many related grammar-based tasks is not developmentally plausible, but there are several ways in which the greater principle—that systematicity can be honed through incentive and practice—has developmental merit. First, children are not born with an adult-like ability to compose functions; in fact, there seem to be important changes between infancy^58^ and pre-school^59^ that could be tied to learning. Second, children become better word learners over the course of development^60^, similar to a meta-learner improving with training. It is possible that children use experience, like in MLC, to hone their skills for learning new words and systematically combining them with familiar words. Beyond natural language, people require a years-long process of education to master other forms of systematic generalization and symbolic reasoning^6,7^, including mathematics, logic and computer programming. Although applying the tools developed here to each domain is a long-term effort, we see genuine promise in meta-learning for understanding the origin of human compositional skills, as well as making the behaviour of modern AI systems more human-like.

## Methods

### Behavioural methods: few-shot learning task

The meaning of each word in the few-shot learning task (Fig. 2) is described as follows (see the ‘Interpretation grammars’ section for formal definitions, and note that the mapping of words to meanings was varied across participants). The four primitive words are direct mappings from one input word to one output symbol (for example, ‘dax’ is RED, ‘wif’ is GREEN, ‘lug’ is BLUE). Each output symbol is a circle of a particular colour. The other three words are functional terms that take arguments. Function 1 (‘fep’ in Fig. 2) takes the preceding primitive as an argument and repeats its output three times (‘dax fep’ is RED RED RED). Function 2 (‘blicket’) takes both the preceding primitive and following primitive as arguments, producing their outputs in a specific alternating sequence (‘wif blicket dax’ is GREEN RED GREEN). Last, function 3 (‘kiki’) takes both the preceding and following strings as input, processes them and concatenates their outputs in reverse order (‘dax kiki lug’ is BLUE RED). We also tested function 3 in cases in which its arguments were generated by the other functions, exploring function composition (‘wif blicket dax kiki lug’ is BLUE GREEN RED GREEN). During the study phase (see description below), participants saw examples that disambiguated the order of function application for the tested compositions (function 3 takes scope over the other functions).

Thirty participants in the United States were recruited using Amazon Mechanical Turk and the psiTurk platform^61^. All of the studies were approved by the NYU IRB, protocol FY2018-1728, and obtained informed consent. The participants were informed that the study investigated how people learn input–output associations, and that they would be asked to learn a set of commands and their corresponding outputs. Learning proceeded in a curriculum with four stages, with each stage featuring both a study phase and a test phase (see Extended Data Fig. 1 for the complete set of study and test instructions). In the first three stages, during the study phase, the participants learned individual functions from just two demonstrations each (functions 1 through 3; Fig. 2a). In the final stage, participants learned to interpret complex instructions by combining these functions (function compositions; Fig. 2a). After all stages, there was a short survey that asked about strategy and any technical problems. Participants spent an average of 23 min in the experiment (minimum 8 min and 41 s; maximum 41 min and 19 s).

Each study phase presented the participants with a set of example input–output mappings. For the first three stages, the study instructions always included the four primitives and two examples of the relevant function, presented together on the screen. For the last stage, the entire set of study instructions was provided together to probe composition. During the study phases, the output sequence for one of the study items was covered and the participants were asked to reproduce it, given their memory and the other items on the screen. Corrective feedback was provided, and the participants cycled through all non-primitive study items until all were produced correctly or three cycles were completed. The test phase asked participants to produce the outputs for novel instructions, with no feedback provided (Extended Data Fig. 1b). The study items remained on the screen for reference, so that performance would reflect generalization in the absence of memory limitations. The study and test items always differed from one another by more than one primitive substitution (except in the function 1 stage, where a single primitive was presented as a novel argument to function 1). Some test items also required reasoning beyond substituting variables and, in particular, understanding longer compositions of functions than were seen in the study phase.

The response interface had a pool of possible output symbols that could be clicked or dragged to the response array. The circles could be rearranged within the array or cleared with a reset button. The study and test set only used four output symbols, but the pool provided six possibilities (that is, there were two extra colours that were not associated to words), to discourage reasoning by exclusion. The assignment of words to colours and functions was randomized for each participant (drawn from nine possible words and six colours), and the first three stages were presented in random order.

We used several strategies to ensure that our participants were paying attention. First, before the experiment, the participants practiced using the response interface and had to pass an instructions quiz; they cycled through the quiz until they passed it. Second, catch trials were included during the test phases, probing the study items rather than new items, with the answers clearly presented on the screen above. There was one catch trial per stage (except the last stage had two); participants were excluded if they missed two or more catch trials (*n* = 5). Finally, query responses were also excluded if the corresponding study phases were not completed correctly (for all items) within three attempts (13% of remaining data).

For statistical analyses of the data from this experiment and elsewhere, we tested for data normalcy and applied alternative nonparametric or permutation tests when the assumptions were not met.

### Interpretation grammars

**Extended Data Fig. 2:**
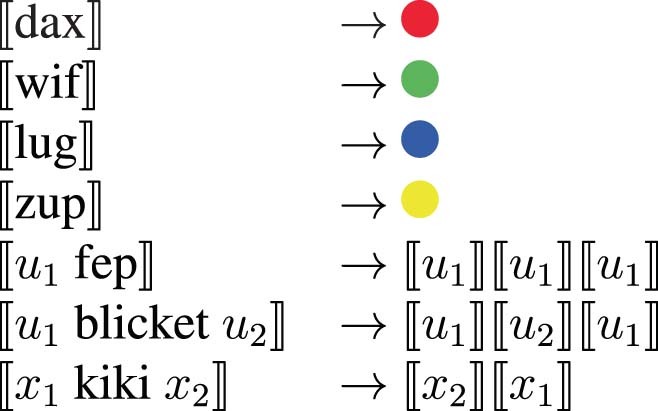
The gold interpretation grammar that defines the human instruction learning task. The double brackets (⟦⟧) denote the interpretation function for translating linguistic instructions into sequences of abstract outputs (colour circles). Each human participant received a different permutation of words and colours. Symbols *x*_*i*_ and *u*_*i*_ denote variables: *x*_*i*_ applies to arbitrary non-empty strings, while *u*_*i*_ applies only to ‘dax’, ‘wif’, ‘lug’, and ‘zup’.

**Extended Data Fig. 3:**
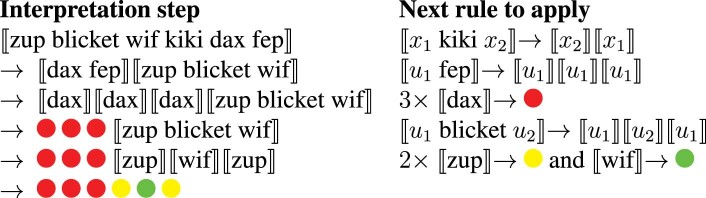
Using the gold interpretation grammar for processing ‘zup blicket wif kiki dax fep’. Each step is annotated with the next re-write rules to be applied, and how many times (e.g., 3 × , since some steps have multiple parallel applications). A rule’s condition is met if and only if it matches the entire string inside the brackets (⟦ ⟧); for instance, only the ‘kiki’ rule applies on the first step because its condition matches two arbitrary non-empty sequences on either side of ‘kiki,’ thus being able to encompass the entire input.

**Extended Data Fig. 4:**
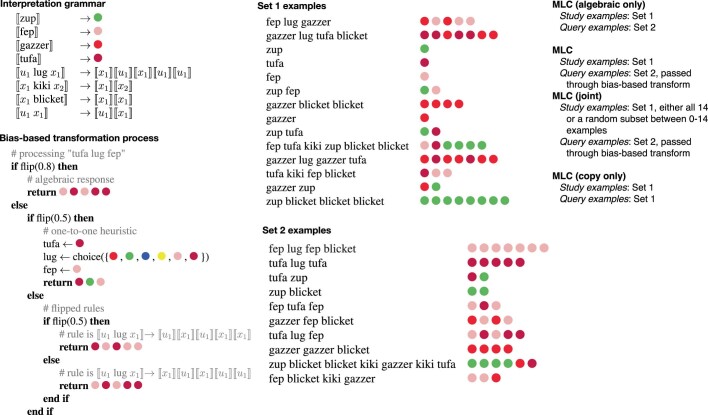
Example meta-learning episode and how it is processed by different MLC variants. The interpretation grammar defines the episode but is not observed directly and must be inferred implicitly. Set 1 has 14 input/output examples consistent with the grammar, used as Study examples for all MLC variants. Set 2 has 10 examples, used as Query examples for most MLC variants (except copy only). Pseudocode for the bias-based transformation process is shown here for the instruction ‘tufa lug fep’. This transformation is applied to the query outputs before MLC and MLC (joint) process them. Here, flip (*p*) is a coin flip that returns *True* with probability *p*.

**Extended Data Fig. 5:**
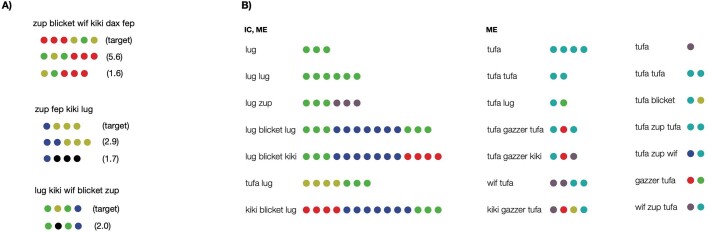
Human responses for the (A) few-shot learning task and (B) open-ended task that most favour MLC (joint) compared to a MLC model optimized for individual tasks only. Panel (A) shows the average log-likelihood advantage for MLC (joint) across five patterns (that is, ll(MLC (joint)) - ll(MLC)), with the algebraic target shown here only as a reference. A black circle indicates a colour that was unused in the study set. Panel (B) shows three participant responses.

The few-shot learning task evaluated with humans and machines is defined through a set of compositional rewrite rules for translating linguistic instructions to output sequences (Extended Data Fig. 2). Inspired by formal semantics^62^, we denote a set of rules such as this as the ‘interpretation grammar’. We refer to the grammar in Extended Data Fig. 2 that defines the human learning task as the ‘gold interpretation grammar’, whereas a different interpretation grammar is shown in Extended Data Fig. 4. The rules apply one by one, based on their conditions, until they produce an output sequence consisting of all terminal symbols (coloured circles). A worked example of interpreting a complex query is shown in Extended Data Fig. 3. Four of the rules define how the primitive words (such as ‘dax’, ‘wif’) map to a single output symbol. The other rules define functions (‘fep’, ‘blicket’ and ‘kiki’) that apply when certain conditions are met through their arguments and, when applied, initiate recursive calls of the interpretation process on their intermediate outputs. Note that a different set of rules will define a different few-shot learning problem; this property is used to define many different few-shot learning problems for optimizing MLC. Although the situation does not arise for the study or query instructions in the few-shot task (see the ‘Behavioural methods: few-shot learning task’ section), it is possible that two rules satisfy their conditions at the same intermediate step. If so, the first rule in the interpretation grammar listing is used in order to resolve the ambiguity.

### Behavioural methods: open-ended task

The instructions were as similar as possible to the few-shot learning task, although there were several important differences. First, because this experiment was designed to probe inductive biases and does not provide any examples to learn from, it was emphasized to the participants that there are multiple reasonable answers and they should provide a reasonable guess. Second, the participants responded to the query instructions all at once, on a single web page, allowing the participants to edit, go back and forth, and maintain consistency across responses. By contrast, the previous experiment collected the query responses one by one and had a curriculum of multiple distinct stages of learning.

Thirty participants in the United States were recruited using Mechanical Turk and psiTurk. The participants produced output sequences for seven novel instructions consisting of five possible words. The participants also approved a summary view of all of their responses before submitting. There were six pool options, and the assignment of words and item order were random. One participant was excluded because they reported using an external aid in a post-test survey. On average, the participants spent 5 min 5 s in the experiment (minimum 2 min 16 s; maximum 11 min 23 s).

### Implementation of MLC

#### Architecture and optimizer

As shown in Fig. 4, our MLC implementation uses a standard seq2seq transformer^26^. This architecture involves two neural networks working together—an encoder transformer to process the query input and study examples, and a decoder transformer to generate the output sequence. Both the encoder and decoder have 3 layers, 8 attention heads per layer, input and hidden embeddings of size 128, and a feedforward hidden size of 512. Following GPT^63^, GELU^64^ activation functions are used instead of ReLU. In total, the architecture has about 1.4 million parameters. Note that an earlier version of memory-based meta-learning for compositional generalization used a more limited and specialized architecture^30,65^.

The encoder network (Fig. 4 (bottom)) processes a concatenated source string that combines the query input sequence along with a set of study examples (input/output sequence pairs). The encoder vocabulary includes the eight words, six abstract outputs (coloured circles), and two special symbols for separating the study examples (∣ and →). The decoder network (Fig. 4 (top)) receives messages from the encoder and generates the output sequence. The decoder vocabulary includes the abstract outputs as well as special symbols for starting and ending sequences (<SOS> and <EOS>, respectively). Sinusoidal positional encodings are added to the input embeddings^26^.

MLC was trained to minimize the cross-entropy loss (averaged over tokens) with the Adam optimizer and a batch size of 25 episodes. Each episode contains many study examples and query examples (for example, up to 14 study examples and 10 queries in optimization for the few-shot learning task) and the effective sequence-level batch size was therefore larger (for example, (14 + 10)25 = 600). Training lasted for 50 epochs. The learning rate was 0.001, with a warm-up applied for the first epoch and then a linear decrease to 0.00005 across training. Dropout of 0.1 was applied to the input embeddings and transformers. For meta-training procedures with a validation set (for example, 200 held-out grammars for few-shot instruction learning), a variant of early stopping was used: although training was not actually truncated, the best parameter setting (across intervals of 100 steps) was saved according to the validation loss. All of the networks were trained using a NVIDIA Titan RTX GPU.

#### Meta-training procedures for MLC and MLC variants

MLC optimizes the transformers for systematic generalization through high-level behavioural guidance and/or direct human behavioural examples. To prepare MLC for the few-shot instruction task, optimization proceeds over a fixed set of 100,000 training episodes and 200 validation episodes. Extended Data Figure 4 illustrates an example training episode and additionally specifies how each MLC variant differs in terms of access to episode information (see right hand side of figure). Each episode constitutes a seq2seq task that is defined through a randomly generated interpretation grammar (see the ‘Interpretation grammars’ section). The grammars are not observed by the networks and must be inferred (implicitly) to successfully solve few-shot learning problems and make algebraic generalizations. The optimization procedures for the MLC variants in Table 1 are described below.

*MLC (algebraic only).* The interpretation grammars that define each episode were randomly generated from a simple meta-grammar. An example episode with input/output examples and corresponding interpretation grammar (see the ‘Interpretation grammars’ section) is shown in Extended Data Fig. 4. Rewrite rules for primitives (first 4 rules in Extended Data Fig. 4) were generated by randomly pairing individual input and output symbols (without replacement). Rewrite rules for defining functions (next 3 rules in Extended Data Fig. 4) were generated by sampling the left-hand sides and right-hand sides for those rules. For the left-hand side (for example, ⟦*u*_1_ lug *x*_1_⟧ for the fifth rule in Extended Data Fig. 4), rules chose an input symbol as function name, whether the function has one or two arguments (with the function name appearing after the argument or in-between arguments, respectively), and whether each argument can take arbitrary non-empty strings (*x*_1_ or *x*_2_) or just the primitive inputs (*u*_1_ or *u*_2_). A rule’s right-hand side was generated as an arbitrary string (length ≤ 8) that mixes and matches the left-hand-side arguments, each of which are recursively evaluated and then concatenated together (for example, ⟦*x*_1_⟧ ⟦*u*_1_⟧ ⟦*x*_1_⟧ ⟦*u*_1_⟧ ⟦*u*_1_⟧). The last rule was the same for each episode and instantiated a form of iconic left-to-right concatenation (Extended Data Fig. 4). Study and query examples (set 1 and 2 in Extended Data Fig. 4) were produced by sampling arbitrary, unique input sequences (length ≤ 8) that can be parsed with the interpretation grammar to produce outputs (length ≤ 8). Output symbols were replaced uniformly at random with a small probability (0.01) to encourage some robustness in the trained decoder. For this variant of MLC training, episodes consisted of a latent grammar based on 4 rules for defining primitives and 3 rules defining functions, 8 possible input symbols, 6 possible output symbols, 14 study examples and 10 query examples. The study examples were presented in shuffled order on each episode.

The validation episodes were defined by new grammars that differ from the training grammars. Grammars were only considered new if they did not match any of the meta-training grammars, even under permutations of how the rules are ordered. The gold interpretation grammar that produced the few-shot instruction-learning task with humans and machines (Extended Data Fig. 2) was also reserved for testing in this way, with an additional structural requirement that grammars for producing the training and validation episodes should also not match the gold grammar through any permutation of the input and output symbol assignments.

For successful optimization, it is also important to pass each study example (input sequence only) as an additional query when training on a particular episode. This effectively introduces an auxiliary copy task—matching the query input sequence to an identical study input sequence, and then reproducing the corresponding study output sequence—that must be solved jointly with the more difficult generalization task.

*M﻿LC for the few-shot instruction-learning task.* Optimization closely followed the procedure outlined above for the algebraic-only MLC variant. The key difference here is that full MLC model used a behaviourally informed meta-learning strategy aimed at capturing both human successes and patterns of error. Using the same meta-training episodes as the purely algebraic variant, each query example was passed through a bias-based transformation process (see Extended Data Fig. 4 for pseudocode) before MLC processed it during meta-training. Specifically, each query was paired with its algebraic output in 80% of cases and a bias-based heuristic in the other 20% of cases (chosen to approximately reflect the measured human accuracy of 80.7%). To create the heuristic query for meta-training, a fair coin was flipped to decide between a stochastic one-to-one translation and a noisy application of the underlying grammatical rules. For the one-to-one translations, first, the study examples in the episode are examined for any instances of isolated primitive mappings (for example, ‘tufa → PURPLE’). Second, each input symbol is mapped superficially to a single output symbol (in a left-to-right manner) using either the corresponding primitive mapping if observed as a study example, or using an arbitrary output symbol if a primitive mapping is not observed (for example, if the input symbol is a function name). For the noisy rule examples, each two-argument function in the interpretation grammar has a 50% chance of flipping the role of its two arguments. For example, as in Extended Data Fig. 4, the rule ⟦*u*_1_ lug *x*_1_⟧ → ⟦*x*_1_⟧ ⟦*u*_1_⟧ ⟦*x*_1_⟧ ⟦*u*_1_⟧ ⟦*u*_1_⟧, when flipped, would be applied as ⟦*u*_1_ lug *x*_1_⟧ → ⟦*u*_1_⟧ ⟦*x*_1_⟧ ⟦*u*_1_⟧ ⟦*x*_1_⟧ ⟦*x*_1_⟧.

*MLC for the open-ended task.* An epoch of optimization consisted of 100,000 episode presentations based on the human behavioural data. To produce one episode, one human participant was randomly selected from the open-ended task, and their output responses were divided arbitrarily into study examples (between 0 and 5), with the remaining responses as query examples. Additional variety was produced by shuffling the order of the study examples, as well as randomly remapping the input and output symbols compared to those in the raw data, without altering the structure of the underlying mapping. The models were trained to completion (no validation set or early stopping).

*MLC (joint).* Optimization for the joint MLC model, tuned jointly for the few-shot instruction and open-ended tasks, proceeded as described in the two paragraphs above; each epoch combined 100,000 episodes of the few-shot instruction learning optimization and 100,000 episodes of the open-ended optimization. Finally, each epoch also included an additional 100,000 episodes as a unifying bridge between the two types of optimization. These bridge episodes revisit the same 100,000 few-shot instruction learning episodes, although with a smaller number of the study examples provided (sampled uniformly from 0 to 14). Thus, for episodes with a small number of study examples chosen (0 to 5, that is, the same range as in the open-ended trials), the model cannot definitively judge the episode type on the basis of the number of study examples. The models were trained to completion (no validation set or early stopping).

MLC (copy only). Optimization for the copy-only model closely followed the procedure for the algebraic-only variant. Critically, this model was trained only on the copy task of identifying which study example is the same as the query example, and then reproducing that study example’s output sequence (see specification in Extended Data Fig. 4; set 1 was used for both study and query examples). It was not trained to handle novel queries that generalize beyond the study set. Thus, the model was trained on the same study examples as MLC, using the same architecture and procedure, but it was not explicitly optimized for compositional generalization.

#### Evaluation procedures

*Few﻿-shot instruction-learning task.* MLC was evaluated on this task in several ways; in each case, MLC responded to this novel task through learned memory-based strategies, as its weights were frozen and not updated further. MLC predicted the best response for each query using greedy decoding, which was compared to the algebraic responses prescribed by the gold interpretation grammar (Extended Data Fig. 2). MLC also predicted a distribution of possible responses; this distribution was evaluated by scoring the log-likelihood of human responses and by comparing samples to human responses. Although the few-shot task was illustrated with a canonical assignment of words and colours (Fig. 2), the assignments of words and colours were randomized for each human participant. Thus, to evaluate MLC comparably, these factors were also randomized. For comparison with the gold grammar or with human behaviour via log-likelihood, performance was averaged over 100 random word/colour assignments. Samples from the model (for example, as shown in Fig. 2 and reported in Extended Data Fig. 1) were based on an arbitrary random assignment that varied for each query instruction, with the number of samples scaled to 10× the number of human participants.

Open-ended task. MLC was evaluated on sampling human-like responses and predicting human responses through log-likelihood scores. Human participants made plausible guesses for how to respond to 7 query instructions (see the ‘Behavioural methods: open-ended task’ section). They responded jointly to all 7 queries on the same web page; as analysed in the main text, people’s predicted word meanings followed strong consistency constraints across the responses. Thus, to model these data, MLC cannot simply answer the queries independently. Instead, MLC factorizes the problem of responding jointly to 7 query inputs *x*_1_, …, *x*_7_ with 7 query outputs *y*_1_, …, *y*_7_ as

1 $$P(\,{y}_{1},\ldots ,{y}_{7}| {x}_{1},\ldots ,{x}_{7})=\mathop{\prod }\limits_{i=1}^{7}P(\,{y}_{i}| {x}_{i},{x}_{ < i},{y}_{ < i}),$$

using (*x*_1_, *y*_1_), …, (*x*_*i*−1_, *y*_*i*−1_) as study examples for responding to query *x*_*i*_ with output *y*_*i*_. Thus, sampling a response for the open-ended task proceeded as follows. First, MLC samples *P*(*y*_1_∣*x*_1_) with no study examples. Second, when sampling *y*_2_ in response to query *x*_2_, the previously sampled (*x*_1_, *y*_1_) is now a study example, and so on. The query ordering was chosen arbitrarily (this was also randomized for human participants).

For scoring a particular human response *y*_1_, …, *y*_7_ by log-likelihood, MLC uses the same factorization as in equation (1). Performance was averaged over 200 passes through the dataset, each episode with different random query orderings as well as word and colour assignments.

### Alternative neural and symbolic models

In addition to the range of MLC variants specified above, the following additional neural and symbolic models were evaluated.

*Lapse model.* All MLC, symbolic and neural models were fit to the human behavioural responses (Table 1) with a lapse parameter *λ*. With this parameter, the probability of a participant producing any given output symbol *s* ∈ *S* is $P(s)=(1-\lambda ){P}_{M}(s)+\lambda \frac{1}{| S| }$, where *S* (with cardinality ∣*S*∣) is the set of abstract outputs (colour circles) plus the end-of-sequence token (<EOS>) and *P*_*M*_ is the model prediction before the lapse mechanism. If the model has no prediction for a particular symbol (for example, this symbol extends beyond the model’s predicted output sequence), $P(s)=\frac{1}{| S| }$.

*Symbolic (oracle).* This probabilistic symbolic model assumes that people can infer the gold grammar from the study examples (Extended Data Fig. 2) and translate query instructions accordingly. Non-algebraic responses must be explained through the generic lapse model (see above), with a fit lapse parameter. Note that all of the models compared in Table 1 have the same opportunity to fit a lapse parameter.

Symbolic (oracle/biases). For the few-shot instruction-learning task, this probabilistic symbolic model augments the oracle, described above, by passing the algebraic input/output pairs through the same bias-based transformation process used when optimizing MLC (see pseudocode in Extended Data Fig. 4 and see the ‘MLC few-shot instruction-learning task’ section for more description). Thus, using the gold grammar in Extended Data Fig. 2, this model predicts a mixture of algebraic outputs, one-to-one translations and noisy rule applications to account for human behaviour.

For the open-ended task, this probabilistic symbolic model operationalizes the three key inductive biases. Using the same factorization as MLC does for the open-ended task (equation (1)), the response sequence *y*_*i*_ to query sequence *x*_*i*_ is modelled based on previous participant responses, *P*(*y*_*i*_∣*x*_*i*_, *x*_<*i*_, *y*_<*i*_). Each input token within the sequence *x*_*i*_ is stochastically translated as a single output token in *y*_*i*_ using a left-to-right (iconic concatenation), one-to-one strategy. For example, if *x*_*i*_ is ‘dax wug’, a coloured circle for ‘dax’ is sampled in proportion to the number of times ‘dax’ aligned with each coloured circle in the previous *x*_<*i*_ and *y*_<*i*_ pairs. After handling ‘dax’, a coloured circle for ‘wug’ is sampled in the same manner. If a word is new (and does not appear previously in *x*_<*i*_), its coloured circle is sampled from the set of unused output symbols (that do not appear in *y*_<*i*_), implementing mutual exclusivity. As with all models, a fit lapse parameter is also used.

Neural (basic seq2seq). A basic seq2seq transformer can be obtained through a straightforward modification of the MLC diagram (Fig. 4): the study examples were excluded from the input sequence, leaving the transformer to process only the query input before producing the query output. Given that only the architecture’s use has changed (not the architecture itself), the model has approximately the same number of learnable parameters as in MLC (except for the smaller input vocabulary). Without access to study examples, the model is poorly equipped for learning words with changing meanings; it has no in-context memory and, therefore, all of its knowledge must be stored in the learned weights. To perform the few-shot instruction-learning task, the basic seq2seq model was trained in the typical way for seq2seq modelling: training iterates over the input/output sequence pairs with the aim of learning the target mapping. In this case, the training set is the 14 study instructions and the test set is the 10 query instructions (Extended Data Fig. 1). Otherwise, the same architecture and optimizer was used as described in the ‘Architecture and optimizer’ section. The network was trained for 1,000 epochs over the batched set of study instructions. It was not clear how much training would be optimal and we wanted to examine this model under favourable conditions. To this end, we gave it an additional advantage not offered to any other model class: we tracked each step of the optimizer and selected the best parameter values on the basis of the test loss. Typically, this point was reached within a few dozen steps. Nevertheless, all 10 runs failed to generalize systematically on the few-shot instruction task (0% exact-match accuracy).

We informally examined a couple of other basic seq2seq variants. First, we evaluated lower-capacity transformers but found that they did not perform better. Second, we tried pretraining the basic seq2seq model on the entire meta-training set that MLC had access to, including the study examples, although without the in-context information to track the changing meanings. Then model was then fine-tuned as described above. On the few-shot instruction task, this improves the test loss marginally, but not accuracy.

### Machine learning benchmarks

#### Handling long in-context sequences

**Extended Data Fig. 6:**
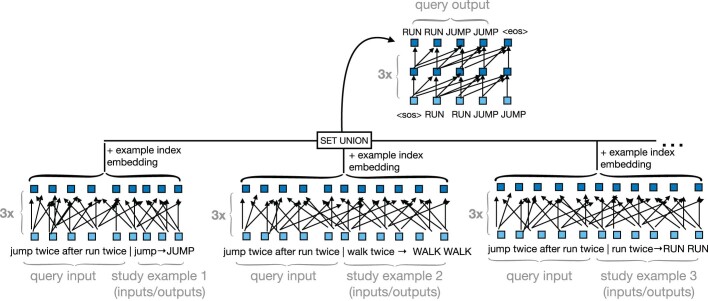
Handling long in-context sequences with a MLC transformer. The query input sequence (shown as ‘jump twice after run twice’) is copied and concatenated to each of the *m* study examples, leading to *m* separate source sequences (3 shown here). A shared standard transformer encoder (bottom) processes each source sequence to produce latent (contextual) embeddings. The contextual embeddings are marked with the index of their study example, combined with a set union to form a single set of source messages, and passed to the decoder. The standard decoder (top) receives this message from the encoder, and then produces the output sequence for the query. Each box is an embedding (vector); input embeddings are light blue and latent embeddings are dark blue.

The tasks from the machine-learning literature that we experimented with, SCAN^11,66^ and COGS^16^, feature long sequences as (in-context) study examples. This raises issues for the previous architecture (see the ‘Architecture and optimizer’ section). Specifically, it is intractable to process a single source sequence that consists of the concatenated query input sequence and multiple study example sequences, which could have a worst-case source sequence of length *S* ≈ 1,500 on COGS and potentially longer in other applications (for each individual study example, the maximum length in SCAN is 9 for inputs and 49 for outputs; the maximum length in COGS is 22 for inputs and 154 for outputs). The bottlenecks are the encoder self-attention layers, which are ${\mathcal{O}}({S}^{2})$. A more scalable procedure for applying a standard transformer (Extended Data Fig. 6) was therefore developed for optimizing MLC on machine learning benchmarks. We copy each query input sequence *m* times and concatenate the copies separately with each of the *m* study examples. This creates *m* smaller source sequences to be processed separately by the standard transformer encoder. Each of the resulting contextual embeddings are then marked according to their origin in one of the *m* study examples, which is done by adding an index embedding vector that enables the decoder to see which embedding came from which study example (one for each index 1, …, *m*). Finally, the set of contextual embeddings is passed to the standard transformer decoder. The decoder cross-attention layers are less expensive, ${\mathcal{O}}(ST)$, because the target sequence length *T*, which does not include any study examples, is typically much shorter (*T* ≪ *S*).

#### Optimization

For each SCAN split, both MLC and basic seq2seq models were optimized for 200 epochs without any early stopping. For COGS, both models were optimized for 300 epochs (also without early stopping), which is slightly more training than the extended amount prescribed in ref. ^67^ for their strong seq2seq baseline. The batch size was 200 episodes for SCAN and 40 episodes for COGS. This more scalable MLC variant, the original MLC architecture (see the ‘Architecture and optimizer’ section) and basic seq2seq all have approximately the same number of learnable parameters (except for the fact that basic seq2seq has a smaller input vocabulary).

Each SCAN episode contained 10 study examples and 2 query examples (COGS used 8 study and 2 query), such that one query example was a randomly chosen study example (as an auxiliary copy task; see the ‘Meta-training procedures for MLC and MLC variants’ section) and the other query was distinct from the study examples and required generalization. All of the query and study examples were drawn from the training corpus. Each episode was scrambled (with probability 0.95) using a simple word type permutation procedure^30,65^, and otherwise was not scrambled (with probability 0.05), meaning that the original training corpus text was used instead. Occasionally skipping the permutations in this way helps to break symmetries that can slow optimization; that is, the association between the input and output primitives is no longer perfectly balanced. Otherwise, all model and optimizer hyperparameters were as described in the ‘Architecture and optimizer’ section.

#### SCAN: meta-training and testing

**Extended Data Fig. 7:**
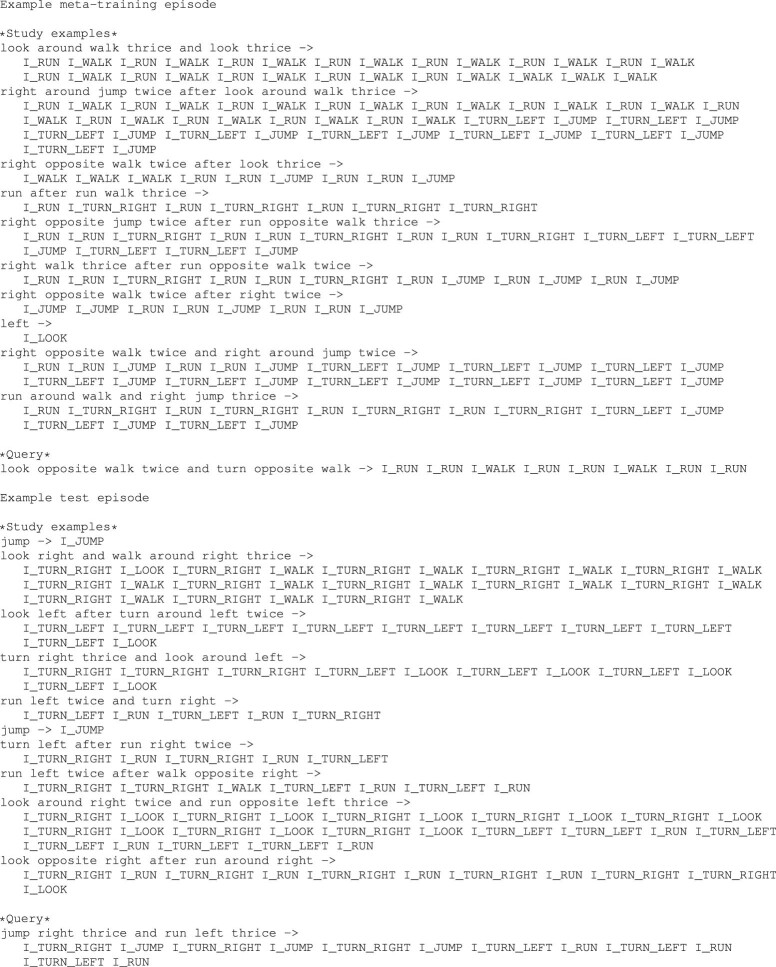
Example SCAN meta-training (top) and test (bottom) episodes for the ‘add jump’ split. The word and action meanings are changing across the meta-training episodes (‘look’, ‘walk’, etc.) and must be inferred from the study examples. During the test episode, the meanings are fixed to the original SCAN forms. Here, the latter probes a compositional use of ‘jump’.

During SCAN meta-training (an example episode is shown in Extended Data Fig. 7), each episode is formed by sampling a set of study and query examples from the training corpus of a particular SCAN split (‘add jump’, ‘around right’ and so on). Given these examples, a simple permutation procedure remaps the full set of output actions (‘JUMP’, ‘RUN’, ‘WALK’, ‘LOOK’, ‘TURN LEFT’, ‘TURN RIGHT’) through a random permutation of these same set of actions, and remaps the input primitives (‘jump’, ‘run’, ‘walk’, ‘look’, ‘left’, ‘right’) through another random permutation to the same set of words. Note that several other input words (the mostly ‘functional’ words ‘turn’, ‘twice’, ‘thrice’, ‘around’, ‘opposite’, ‘and’, ‘after’) have stable meanings that can be stored in the model weights. To make sense of an episode, MLC must become adept at inferring, from just a few study examples, how words map to meanings. MLC must also become adept at composition: it must systematically compose the inferred word meanings to correctly answer the queries.

During SCAN testing (an example episode is shown in Extended Data Fig. 7), MLC is evaluated on each query in the test corpus. For each query, 10 study examples are again sampled uniformly from the training corpus (using the test corpus for study examples would inadvertently leak test information). Neither the study nor query examples are remapped; in other words, the model is asked to infer the original meanings. Finally, for the ‘add jump’ split, one study example is fixed to be ‘jump → JUMP’, ensuring that MLC has access to the basic meaning before attempting compositional uses of ‘jump’.

#### COGS: meta-training and testing

**Extended Data Fig. 8:**
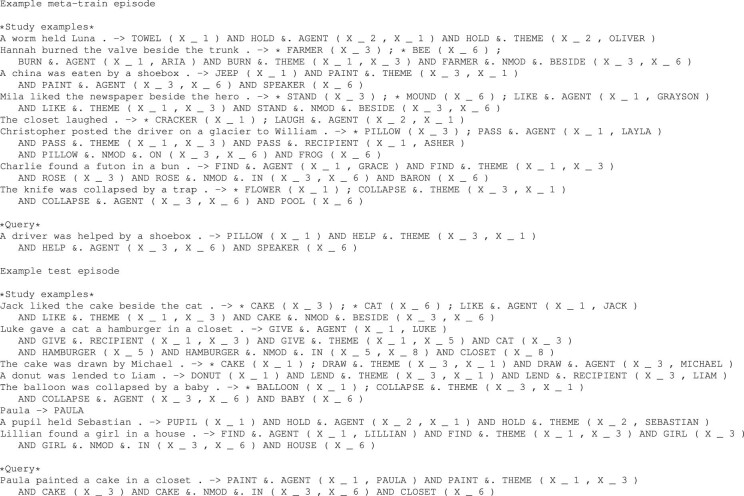
Example COGS meta-training (top) and test (bottom) episodes. Word meanings are changing across the meta-training episodes (here, ‘driver’ means ‘PILLOW’, ‘shoebox’ means ‘SPEAKER’ etc.) and must be inferred from the study examples. The meanings are fixed to the original forms during the test episode. This test episode probes the understanding of ‘Paula’ (proper noun), which just occurs in one of COGS’s original training patterns.

The COGS output expressions were converted to uppercase to remove any incidental overlap between input and output token indices (which MLC, but not basic seq2seq, could exploit). As in SCAN meta-training, an episode of COGS meta-training involves sampling a set of study and query examples from the training corpus (see the example episode in Extended Data Fig. 8). The vocabulary in COGS is much larger than in SCAN; thus, the study examples cannot be sampled arbitrarily with any reasonable hope that they would inform the query of interest. Instead, for each vocabulary word that takes a permuted meaning in an episode, the meta-training procedure chooses one arbitrary study example that also uses that word, providing the network an opportunity to infer its meaning. Any remaining study examples needed to reach a total of 8 are sampled arbitrarily from the training corpus.

COGS is a multi-faceted benchmark that evaluates many forms of systematic generalization. To master the lexical generalization splits, the meta-training procedure targets several lexical classes that participate in particularly challenging compositional generalizations. As in SCAN, the main tool used for meta-learning is a surface-level token permutation that induces changing word meaning across episodes. These permutations are applied within several lexical classes; for examples, 406 input word types categorized as common nouns (‘baby’, ‘backpack’ and so on) are remapped to the same set of 406 types. The other remapped lexical classes include proper nouns (103 input word types; ‘Abigail’, ‘Addison’ and so on), dative verbs (22 input word types; ‘given’, ‘lended’ and so on) and verbs in their infinitive form (21 input word types; such as ‘walk’, ‘run’). Surface-level word type permutations are also applied to the same classes of output word types. Other verbs, punctuation and logical symbols have stable meanings that can be stored in the model weights. Importantly, although the broad classes are assumed and could plausibly arise through simple distributional learning^68,69^, the correspondence between input and output word types is unknown and not used.

In one case, COGS meta-learning goes beyond surface-level remapping to use a minimal amount of semantic structure. To guide the networks toward flexible substitution of common nouns with proper nouns, any common noun input token has an independent chance of replacement (probability 0.01) with an arbitrary proper noun input token, while also removing the preceding determiner token. Independently, any common noun output token can also be arbitrarily remapped (again with probability 0.01) to a proper noun output token, with the corresponding minimal change to the structural form to remove the determiner (if remapping the output token ‘cookie’ to ‘John’, the cookie(*x*_*i*_) predicate is removed, occurrences of variable *x*_*i*_ are replaced with ‘John’ and variables *j* > *i* are decremented by 1). As before, the correspondence between input and output tokens is unknown, both at the levels of a sentence and the whole dataset. Thus, during an episode of meta-training, a common noun (phrase) might correspond to a logical form expressing a proper noun or vice versa. At test, MLC must sort this out and recover how proper and common nouns work on the basis of the study examples.

During the COGS test (an example episode is shown in Extended Data Fig. 8), MLC is evaluated on each query in the test corpus. For each query, eight study examples are sampled from the training corpus, using the same procedure as above for picking study examples that facilitate word overlap (note that picking study examples from the generalization corpus would inadvertently leak test information). Neither the study nor query examples are remapped to probe how models infer the original meanings.

### Reporting summary

Further information on research design is available in the Nature Portfolio Reporting Summary linked to this article.

## Online content

Any methods, additional references, Nature Portfolio reporting summaries, source data, extended data, supplementary information, acknowledgements, peer review information; details of author contributions and competing interests; and statements of data and code availability are available at 10.1038/s41586-023-06668-3.

### Supplementary information

Supplementary Information Supplementary 1–3 (additional modelling results, experiment probing additional nuances in inductive biases, and few-shot instruction learning with OpenAI models), Supplementary Figs. 1–7 and Supplementary References.

Reporting Summary

## Data Availability

Human behavioural data are available at Zenodo (10.5281/zenodo.8274609). The complete set of human and machine responses is also illustrated and viewable in HTML at the previous link. The human behavioural data also appeared in a previous non-archival conference paper^70^.

## Code availability

MLC source code and pretrained models are available online^71^, including MLC models of human behaviour (10.5281/zenodo.8274609) and MLC models applied to machine learning benchmarks (10.5281/zenodo.8274617). Any additional code is available on request.
