# Supplementary material for: Human-like systematic generalization through a meta-learning neural network
Source: Nature. 2023 Oct 25;623(7985):115–21. doi: 10.1038/s41586-023-06668-3 (PMC10620072; doi:10.1038/s41586-023-06668-3)
Supplement: Supplementary file 1 — Supplementary 1–3 (additional modelling results, experiment probing additional nuances in inductive biases, and few-shot instruction learning with OpenAI models), Supplementary Figs. 1–7 and Supplementary References. [file 41586_2023_6668_MOESM1_ESM.pdf]

nature portfolio

https://doi.org/10.1038/s41586-023-06668-3

Supplementary information

Human-like systematic generalization through a meta-learning neural network

In the format provided by the authors and unedited

Supplementary Information: Human-like systematic generalization through a meta-learning neural network

Brenden M. Lake[1*] and **Marco Baroni**[2,3]

[1]Department of Psychology and Center for Data Science, New York University
[2]Catalan Institution for Research and Advanced Studies (ICREA)
[3]Department of Translation and Language Sciences, Universitat Pompeu Fabra

[*]To whom correspondence should be addressed; E-mail: brenden@nyu.edu

Supplementary Information Guide

SI-1 Additional modeling results **S3**

SI-1.1 Few-shot instruction learning task S3

SI-1.2 Learning novel rules S4

SI-1.3 Open-ended task S5

SI-2 Experiment probing additional nuances in inductive biases **S6**

SI-2.1 Behavioral methods S6

SI-2.2 Behavioral results S7

SI-2.3 Modeling methods S10

SI-2.4 Modeling results S10

SI-3 Few-shot instruction learning with OpenAI models **S11**

SI-1 Additional modeling results

SI-1.1 Few-shot instruction learning task

MLC optimization was run 10 times with different random initializations and other random factors (episode order, study example order, heuristic outputs, etc.). Not every run optimizes successfully, but top-performing runs can be identified through their loss on the prescribed grammatical outputs in the few-shot instruction task (Extended Data Fig. 2) or their validation loss. Even without selecting the most algebraic model, a typical MLC run has strong algebraic capabilities, achieving a mean of 92.9% ($SD = 8.2$) exact match accuracy on the key few-shot instruction task and a mean of 95.3% ($SD = 0.4$) on the validation episodes. A typical MLC (algebraic only) run also achieved high accuracy on the few-shot task ($M = 93.6\%, SD = 9.0$) and validation episodes ($M = 94.7\%, SD = 2.4$) (see the "Behavioural methods: few-shot learning task" section of the Methods for a description of the MLC variants). Similarly, a typical MLC (joint) run also performed well on the few-shot task ($M = 96.8\%, SD = 5.2$) and the algebraic-based validation episodes ($M = 95.8\%, SD = 0.4$). Note that MLC (joint) was run 15 times (5-fold cross-validation on open-ended task with 3 runs replicating each split).

Beyond predicting the gold output sequences, the variability across runs for predicting human behavior is shown in Fig. SI-1. When using the most algebraic run or average run, MLC (joint) is the strongest model for predicting human behavior. But it is less straightforward to determine if MLC (joint) is reliably better than MLC for arbitrary runs, as the 15 runs for MLC (joint) cycle through 5 different splits of the open-ended task. Ignoring this factor in a Mann-Whitney U test (two-sided, exact), there is a trend toward better average performance for MLC (joint) although it is marginal ($U = 109.0$, $p = 0.062$, Cohen's $d = 0.69$). Extended Data Fig. 5A shows the top-five human responses for which MLC (joint) provides the largest predictive advantage. The additional open-ended training provides advantage in predicting responses

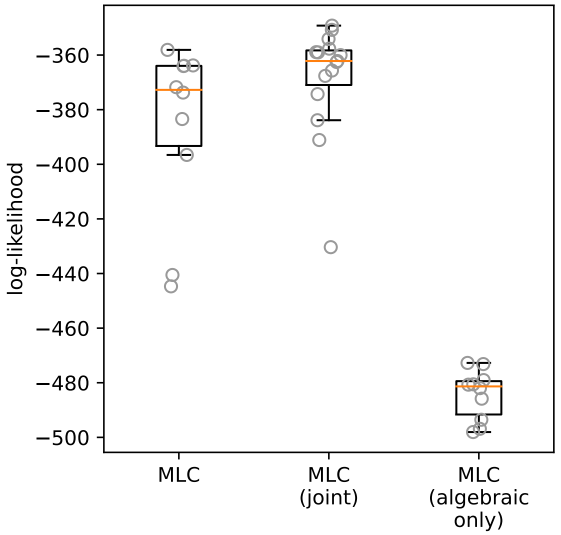

Figure SI-1: Variability across runs in model log-likelihood of human few-shot learning behavior. All data points are shown in grey. The box plot shows the median, first quartile, and third quartile; the whiskers extend from the box by 1.5x the inter-quartile range. There were $n = 10$ runs for MLC and MLC (algebraic only) and $n = 15$ runs for MLC (joint). A Mann-Whitney U test comparing MLC vs. MLC (joint) results in $U = 109.0$, $p = 0.062$, Cohen's $d = 0.69$.

that use colors outside the study set (black circles), which is characteristic of the open-ended task. Additionally, these patterns tend to have added or dropped output symbols compared to the algebraic standards; similarly, participants in the open-ended task were more flexible in the number of output symbols they map to an input symbol.

SI-1.2 Learning novel rules

MLC was evaluated on its ability to infer novel rules. Here, we define novel to mean that no semantically equivalent rules were used to produce one of the meta-training episodes (regardless of rule name, or interchangeable use of variables u_1, u_2, x_1, x_2; see the "Meta-training procedures for MLC and MLC variants" section of the Methods for the details of the interpretation grammar formulation).[1] Thus, any capabilities that MLC has for inferring and processing

[1]For instance, if $[\![u_1 \text{ fep } x_1]\!] \rightarrow [\![x_1]\!][\![x_1]\!][\![x_1]\!][\![u_1]\!][\![x_1]\!][\![x_1]\!][\![u_1]\!][\![x_1]\!]$ was used to create a meta-training episode, then $[\![u_1 \text{ wif } u_2]\!] \rightarrow [\![u_2]\!][\![u_2]\!][\![u_2]\!][\![u_1]\!][\![u_2]\!][\![u_2]\!][\![u_1]\!][\![u_2]\!]$ could not be a test rule. The fact they use a different name

novel rules would need to arise through the knowledge embedded in its frozen weights, as no episode-specific changes are allowed during testing.

As described in the "Meta-training procedures for MLC and MLC variants" section of the Methods, the MLC models were optimized for few-shot instruction learning across 100K training episodes. To evaluate the acquisition of novel rules, we identified 26 rules that were not used in generating these episodes. New test episodes (130 total; 5 replications per rule) were generated from the meta-training distribution of grammars but with one of the rules fixed to be novel. The novel rules each had the maximum possible length (8) of right-hand-side variable occurrences. For example one novel rule was $[\![u_1 \text{ fep } u_2]\!] \rightarrow [\![u_2]\!][\![u_2]\!][\![u_2]\!][\![u_1]\!][\![u_2]\!][\![u_2]\!][\![u_1]\!][\![u_2]\!]$ such that the rule name ("fep") varies over the 5 replications. For each episode, the query instructions consists of the novel rule applied to all combinations of primitives, e.g., "dax fep blicket', "dax fep zup" for all primitives in the episodes. In other episodes, the novel rules and associated query examples take a similar form.

MLC succeeds at acquiring these novel rules. Each of the three most capable MLC models (main text Table 1) achieved over 99% exact-match accuracy on inferring and applying these new rules. Specifically, MLC achieves 99.3%, MLC (joint) achieves 99.8%, and MLC (algebraic only) achieves 99.4%.

SI-1.3 Open-ended task

MLC optimization was run 3 times for each of the 5-fold cross-validation splits. Examining the total held-out log-likelihood across these 15 simulations, a two-way ANOVA revealed that MLC (joint) is better than plain MLC for predicting held-out human participants in the open-ended task ($F(1, 24) = 8.0,\ p < 0.01$), when controlling for the particular cross-validation split ($F(4, 24) = 185.7,\ p < 0.0001$). This indicates a benefit for training MLC jointly on

is irrelevant and they also apply to the same set of cases because 8 is the maximum output length for a command.

the few-shot instruction and open-ended tasks. Extended Data Fig. 5B shows the three human participants best predicted by MLC (joint) relative to MLC. These participants tended not to follow the key inductive biases; in fact, the rightmost two patterns, if interpreted with respect to algebraic rules, would demand complex functions. The leftmost pattern assigns one color per letter, which is a hypothesis that no MLC model considers as words are represented as arbitrary tokens.

SI-2 Experiment probing additional nuances in inductive biases

Here we report a supplemental study investigating additional nuances in human inductive biases. We suspect that people's inductive biases may be further nuanced in ways that the main experiments did not explore, further challenging not only symbolic accounts but also the previous MLC transformers. To examine the limits of the models, human participants were probed regarding additional contextual factors related to the inductive biases. The complete set of human and model responses is viewable on the web (see main text Data availability).

SI-2.1 Behavioral methods

Twenty-eight participants in the United States were recruited using Mechanical Turk and psiTurk. The instructions were as similar as possible to the previous experiments (see the "Behavioural methods: few-shot learning task" and "Behavioural methods: open-ended task" sections of the Methods). There were 14 trials that evaluated biases under different circumstances. Participants were asked to consider each trial independently. Each trial provided a set of study instructions (input-output mappings) and asked participants to make a judgment about a single query instruction. To highlight the independence between trials, the word and colors were re-randomized for each trial from a larger set of 20 possible words and 8 colors. As in the open-

ended task, participants were told that there were multiple reasonable answers for a given trial and were instructed to provide a reasonable guess. Two catch trials used query instructions that were identical to a study instruction. Missing a catch trial was the only criterion for exclusion ($N = 6$). There was no memory quiz for the study examples since each contained just a few instructions. On average, participants spent 8 minutes 47 seconds in the experiment (minimum 3 minutes 33 seconds; maximum 28 minutes 31 seconds).

Six trials probed people's sensitivity to different aspects of mutual exclusivity, specifically the amount of counter-evidence (either 0, 1, vs. 2 counter examples; see Fig. SI-2A&B for examples) or the number of output options (2 vs. 6). Three trials probed iconic concatenation, specifically in a case that violates one-to-one (one word maps to a sequence of two or three outputs; Fig. SI-2C). Lastly, three trials probed how people weigh ME versus one-to-one when following both biases is not possible. The design aimed to minimize the risk that the biases could be learned from the stimuli themselves. None of the study instructions demonstrated how to concatenate, facilitating a pure evaluation of concatenation preferences. Across the test trials, the study instructions contained examples both consistent with ME, and examples inconsistent with ME. The design did not explicitly control for the one-to-one bias.

SI-2.2 Behavioral results

There was strong evidence for each of the three inductive biases. The classic mutual exclusivity (ME) effect [1] was replicated within our seq2seq learning paradigm. If "dax" means ●, what is a "zup"? As shown in the top-left cell of Fig. SI-2A, most participants (18 of 22; 81.8%) chose a single ● symbol as their response if the pool provided only ● and ● as options, and a larger fraction (20 of 22; 90.9%) followed ME by choosing a (possibly multi-element) meaning different from ●.

Although the ME effect was robust, interestingly, it was sensitive to context and was not

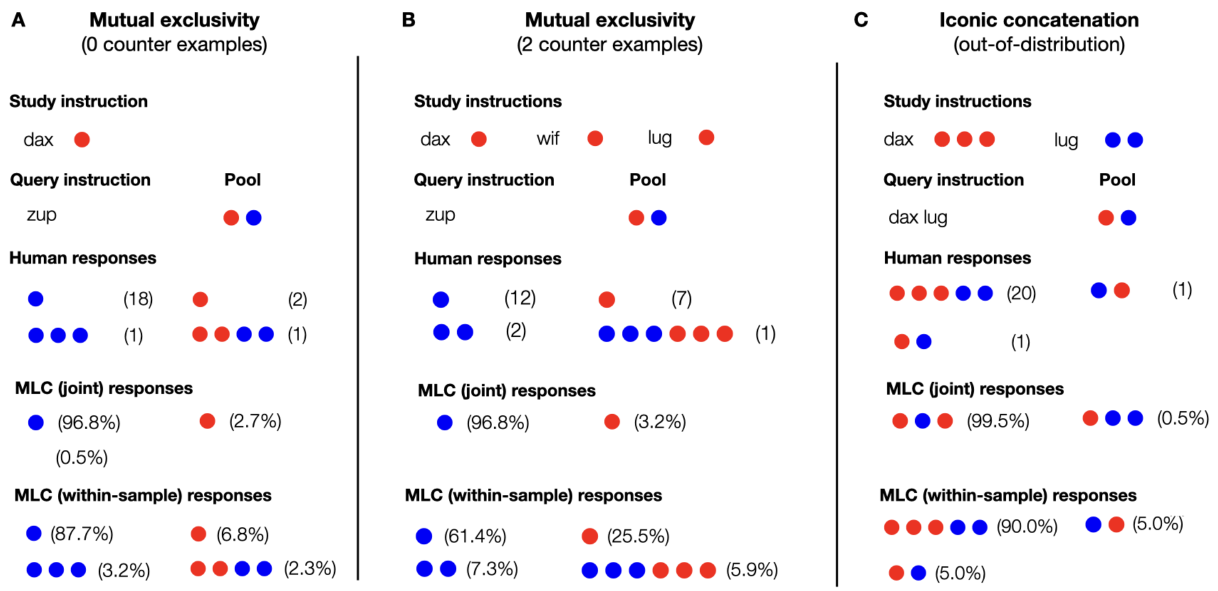

Figure SI-2: Additional nuance in the inductive biases. Participants were asked to respond to the Query instruction given the Study instructions, using only the output symbols in the pool. The mutual exclusivity trials shown had either 0 (A) or 2 (B) counter-examples, with only 2 allowed options in the pool. Participant responses are marked with count in parentheses, and MLC is marked with percent of samples in parentheses. A canonical word and color assignment is shown here although it was randomized for participants.

rigidly applied. The other ME trials examined the influence of two additional factors (Fig. SI-3 for summary; left plot): the number of contradictory examples provided (0–2; Fig. SI-2A vs. B) and the number of output symbols available in the response pool (2 vs. 6). With these two variables as fixed effects, we fit a logistic mixed model predicting whether people produced a novel (non-study) output sequence for each, i.e., if the response was consistent with ME (y $\sim$ n_contra_examples + pool_size + (1 | participant_id), with pool_size as categorical). The percent of all responses consistent with ME is 68.2% ($N = 132$). Both the number of contradictory examples ($\beta = 1.76$, $SE = 0.483$, $Z = 3.64$, $p < 0.001$) and pool size ($\beta = 2.05$, $SE = 0.698$, $Z = 2.93$, $p < 0.01$) were significant predictors of which particular responses were ME-consistent, indicating that people were willing to override or weaken ME when faced with more

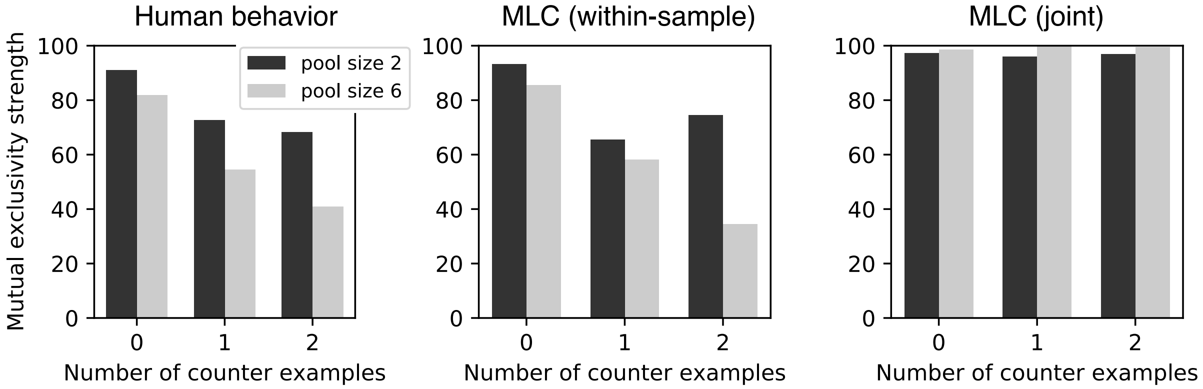

Figure SI-3: Mutual exclusivity (ME) strength in humans and models. For people and the MLC (within-sample) fit, the proportion of responses consistent with ME (y-axis) largely declines with the number of contradictory examples and the number of output symbols available in the response pool. For MLC (joint), ME is strong and absolute.

ME counter-evidence (or equivalently in our case, positive evidence that ● is the right answer), or when more output symbols were available in the pool (Fig. SI-3; left plot). The second effect is intriguing. Although we leave a detailed analysis to future work, we conjecture that it stems from pragmatic reasoning on behalf of the participants: When five yet-to-be-named objects are in the pool, ME is such a weak heuristic that participants might conclude that the experiment is not asking them to rely on it.

There was strong confirmatory evidence for iconic concatenation. Across three trials that examined this bias in various forms, we found that 93.9% ($N = 66$) of responses were consistent with iconic concatenation, even though no examples of concatenation were provided during this experiment (see example in Fig. SI-2C). In three trials where all of the output symbols in the pool were already assigned to unique words, participants had to choose between violating ME by reassigning an output symbol, or violating one-to-one by choosing a more complex functional or multi-element meaning. Interestingly, the responses were evenly split (50.0%, $N = 66$) between following one principle versus the other.

SI-2.3 Modeling methods

We simulated out-of-distribution predictions from MLC-joint (see the "Meta-training procedures for MLC and MLC variants" section of the Methods). This model was trained on the few-shot instruction and open-ended tasks, but was not adapted or optimized for this task examining additional nuances. In fact, this model had no mechanism for adapting its predictions based on the available pool of colors, beyond simply masking out unused options, making this, by construction, an extremely unfavorable setting for it.

Additional simulations examined within-distribution predictions from MLC, denoted as MLC (within-sample). The purpose was to see if MLC can capture the full range of behavioral phenomena studied above when optimized within-sample with the aim of capturing all of the training patterns; however, this model should not be credited with novel predictions. Thus, MLC was optimized across the participants ($N = 22$) using the same architecture and optimizer as previous experiments (see the "Architecture and optimizer" section of the Methods). Each epoch constituted 100K passes through the data using randomized words and colors. Differing from the other experiments, the pool of available output options was indicated to the transformer via the source sequence, using a special study example (e.g., '[] $\rightarrow$ ●● ' to indicate pool-size 2 with only red and blue options). The optimizer was run 3 times and the run with the lowest training loss was chosen. MLC (within-sample) vs. human samples can then be compared.

SI-2.4 Modeling results

Fig. SI-2 shows sample responses from the models. MLC-joint (not re-optimized on any new data) demonstrates a strong ME bias for assigning novel meanings to novel words—which is notable since neural networks typically show an anti-ME bias [2]—but in a more rigid and absolute form than people, applying the bias consistently despite these newly introduced factors (in 98.0% of MLC samples compared to 68.2% of human responses; detailed in Fig. SI-3).

People can also flexibly handle new kinds of mappings, such as those that violate one-to-one, while the previous transformer struggles with kinds of mappings it was not exposed to during meta-learning (Fig. SI-2C).

Samples from MLC (within-sample) are a close recapitulation of the human response distribution (using 220 samples per trial). The percent of all samples consistent with ME was 68.6% for MLC (within-sample) and 98.0% for MLC (joint), compared to people's 68.2%. MLC (within-sample) is able to capture the nuance in human ME due to the number of contrary examples and pool size (Fig. SI-3 middle), while MLC (joint) has a very strong ME effect that is absolute and not nuanced (Fig. SI-3 right). For the iconic concatenation probes, the percent of samples following this bias was 93.8% for MLC (within-sample) and 66.7% for MLC (joint), compared to 93.9% for people. For trading off between ME and one-to-one, the percent of responses favoring one-to-one was 53.2% for MLC (within-sample) and 56.2% for MLC (joint), compared to 50.0% for people. Overall, these findings highlight MLC's strengths as well as its limitations: it can capture subtle nuances in human behavior, but it must be optimized for the kinds of generalizations it will be asked to make.

SI-3 Few-shot instruction learning with OpenAI models

We tested if recent pre-trained language models from OpenAI (`https://platform.openai.com/docs/models`) could solve our few-shot instruction learning task (see main text Figure 2 and "Behavioural methods: few-shot learning task" and "Interpretation grammars" sections of the Methods). We examined the strongest models available to us (at time of writing) including the recently released GPT-4 (via the Chat Completion API) and GPT-3.5 (via the Completion API; `text-davinci-003`).

There are many considerations when evaluating these language models, which were designed to have relatively general-purpose capabilities compared to MLC's more specialist ca-

pabilities. We considered the following in our experiments:

- *Chat vs. completion API.* See Fig. SI-5 for an example prompt for the chat API and Fig. SI-6 for the completion API.
- *Batched vs. individualized queries.* After providing the 14 study examples, all 10 queries can be asked at once ("batched"; see Fig. SI-4) or as 10 individual questions, each using a separate prompt ("individualized"; see Fig. SI-5).
- *System prompt.* For the Chat API, we experimented with adding an initial system prompt, "You are a helpful assistant.", although it did not substantially change the results and thus we excluded it.
- *Random vs. sorted order.* The study and query examples were either provided in random order or provided in a sorted order, from shortest-to-longest based on the length of the output sequence. Note that when batched, the sorted order is an especially generous setup: it leaks some information about the expected length of the output sequences at test.
- *Additional training episodes.* The few-shot instruction task was either evaluated directly (Fig. SI-4) or after five additional episodes, from the MLC meta-learning set, were provided in the prompt (Fig. SI-7).

The experiments were run 10 times with different random assignments of the words and colors. Temperature was set at 0 for partial reproducibility (note still that identical API calls do not produce deterministic outputs). The maximum output length was set to 256 tokens for batch prompts and 32 tokens for individual prompts.

We found the best results using GPT-4 (Chat API) with batched queries, sorted examples, and no additional training episodes, achieving 58.0% correct on average over the 10 replications

```
Here are 14 inputs and their corresponding outputs:
dax -> YELLOW
fep -> RED
blicket -> PINK
gazzer -> GREEN
blicket kiki fep -> RED PINK
dax kiki blicket -> PINK YELLOW
blicket wif -> PINK PINK PINK
blicket zup fep -> PINK RED PINK
dax wif -> YELLOW YELLOW YELLOW
fep zup dax -> RED YELLOW RED
blicket wif kiki fep -> RED PINK PINK PINK
fep kiki dax zup blicket -> YELLOW PINK YELLOW RED
blicket kiki fep wif -> RED RED RED PINK
fep zup dax kiki blicket -> PINK RED YELLOW RED

What are the outputs for each of these inputs?
fep kiki gazzer -> ?
gazzer kiki dax -> ?
gazzer wif -> ?
dax zup gazzer -> ?
gazzer zup blicket -> ?
blicket kiki fep zup gazzer -> ?
gazzer wif kiki blicket -> ?
fep kiki gazzer wif -> ?
gazzer zup gazzer kiki gazzer wif -> ?
gazzer zup fep kiki dax wif -> ?
```

Figure SI-4: Example of how the study examples and the query instructions are passed to GPT-4 in the "batched" condition (using the API's "user" role in Chat mode). Here, the study and query examples are in shortest-to-longest order based on length of the output sequence, as opposed to random order, where sorted order improves performance.

($SD = 14.0$). When individualized queries were used instead, performance dropped to 39.0% correct ($SD = 3.2$). Most notably, when the study and query examples were presented in a random order (while still batched), performance dropped dramatically to 14.0% correct ($SD = 19.0$).

It is also interesting that providing five training episodes did not improve performance. The best results we achieved in this setting were 33.0% correct ($SD = 17.7$). We found similar results whether we presented the training episodes as a back-and-forth conversation between a "user" and an "assistant" (Fig. SI-7) or as a long statement from the user.

We found generally worse results with GPT-3.5. When the study examples were in sorted order, the model achieved 27% correct on average over the 10 replications ($SD = 7.8$). When

```
Here are 14 inputs and their corresponding outputs:
dax -> YELLOW
fep -> RED
blicket -> PINK
gazzer -> GREEN
blicket kiki fep -> RED PINK
dax kiki blicket -> PINK YELLOW
blicket wif -> PINK PINK PINK
blicket zup fep -> PINK RED PINK
dax wif -> YELLOW YELLOW YELLOW
fep zup dax -> RED YELLOW RED
blicket wif kiki fep -> RED PINK PINK PINK
fep kiki dax zup blicket -> YELLOW PINK YELLOW RED
blicket kiki fep wif -> RED RED RED PINK
fep zup dax kiki blicket -> PINK RED YELLOW RED

What is the output for this input?
fep kiki gazzer ->
```

Figure SI-5: Example of how the study examples and a query instruction are passed to GPT-4 in the "individualized" condition (with the API's "user" role in Chat mode). Here, the study examples are in shortest-to-longest order based on length of the output sequence, as opposed to random order, where sorted order improves performance.

the study examples were in random order, the model achieved 17% correct on average ($SD = 6.4$).

We conclude that the best pre-trained language models available can, under certain experimental settings, perform reasonably well (58% correct) on our task, especially given their generalist capabilities. On the other hand, this level of performance was well-below the average human participant (80.7%) and the average MLC model run (between 92.9% and 96.8%, depending on MLC variant, see Section SI-1). We also find that GPT performance is fragile: minor differences in how examples are ordered or batched into prompts can lead to dramatic drops in performance, e.g., dropping from 58% to 14% for randomizing the example order. The best language models are still far from perfectly systematic with respect to our task, at least when not directly optimized for it through supervised fine-tuning or advanced prompt engineering.

It is possible that more sophisticated prompting techniques or future models will further improve systematic generalization in large language models. We do not view this possibility as devaluing our findings. First, it's been known since at least GPT-2 [3] that large language

```
dax -> YELLOW
fep -> RED
blicket -> PINK
gazzer -> GREEN
blicket kiki fep -> RED PINK
dax kiki blicket -> PINK YELLOW
blicket wif -> PINK PINK PINK
blicket zup fep -> PINK RED PINK
dax wif -> YELLOW YELLOW YELLOW
fep zup dax -> RED YELLOW RED
blicket wif kiki fep -> RED PINK PINK PINK
fep kiki dax zup blicket -> YELLOW PINK YELLOW RED
blicket kiki fep wif -> RED RED RED PINK
fep zup dax kiki blicket -> PINK RED YELLOW RED
fep kiki gazzer ->
```

Figure SI-6: Example of how the study examples and the query instruction (last line) are passed to GPT-3.5 in the "completion" condition.

models possess some skills for meta-learning and zero-shot generalization; it's conceivable these skills will continue to strengthen at scale [4, 5] and begin to approach or emulate MLC-like targeted systematicity training. Second, a targeted model such MLC, by directly incentivizing systematicity in a relatively small-scale setup, can clarify what exactly is needed for achieving human-like systematic generalization on challenging tasks.

```
> USER:
Problem 1 examples: Here are 14 inputs and their corresponding outputs:
gazzer -> YELLOW
kiki -> BLUE
tufa fep kiki -> BLUE PURPLE
tufa fep gazzer -> YELLOW PURPLE
wif fep wif -> GREEN GREEN
kiki blicket -> BLUE BLUE
kiki fep tufa -> PURPLE BLUE
gazzer fep wif -> GREEN YELLOW
wif gazzer fep tufa -> GREEN PURPLE YELLOW
gazzer tufa kiki -> YELLOW PURPLE BLUE
gazzer fep gazzer blicket -> YELLOW YELLOW YELLOW YELLOW
gazzer tufa tufa blicket -> YELLOW PURPLE PURPLE YELLOW PURPLE PURPLE
tufa tufa fep wif blicket -> PURPLE GREEN PURPLE PURPLE GREEN PURPLE
kiki tufa zup -> BLUE PURPLE BLUE PURPLE BLUE PURPLE

Problem 1 quiz: What are the outputs for each of these inputs?
wif -> ?
tufa -> ?
wif fep kiki -> ?
kiki fep gazzer -> ?
gazzer blicket blicket -> ?
gazzer fep kiki blicket -> ?
kiki blicket zup -> ?
wif fep gazzer zup -> ?
gazzer kiki fep wif blicket -> ?
wif blicket zup -> ?

> ASSISTANT:
Problem 1 quiz: What are the outputs for each of these inputs?
wif -> GREEN
tufa -> PURPLE
wif fep kiki -> BLUE GREEN
kiki fep gazzer -> YELLOW BLUE
gazzer blicket blicket -> YELLOW YELLOW YELLOW YELLOW
gazzer fep kiki blicket -> BLUE YELLOW BLUE YELLOW
kiki blicket zup -> BLUE BLUE BLUE BLUE BLUE BLUE
wif fep gazzer zup -> YELLOW GREEN YELLOW GREEN YELLOW GREEN
gazzer kiki fep wif blicket -> YELLOW GREEN BLUE YELLOW GREEN BLUE
wif blicket zup -> GREEN GREEN GREEN GREEN GREEN GREEN

...

> USER:
Problem 6 examples: Here are 14 inputs and their corresponding outputs:
dax -> YELLOW
fep -> RED
blicket -> PINK
gazzer -> GREEN
blicket kiki fep -> RED PINK
dax kiki blicket -> PINK YELLOW
blicket wif -> PINK PINK PINK
blicket zup fep -> PINK RED PINK
dax wif -> YELLOW YELLOW YELLOW
fep zup dax -> RED YELLOW RED
blicket wif kiki fep -> RED PINK PINK PINK
fep kiki dax zup blicket -> YELLOW PINK YELLOW RED
blicket kiki fep wif -> RED RED RED PINK
fep zup dax kiki blicket -> PINK RED YELLOW RED

Problem 6 quiz: What are the outputs for each of these inputs?
fep kiki gazzer -> ?
gazzer kiki dax -> ?
gazzer wif -> ?
dax zup gazzer -> ?
gazzer zup blicket -> ?
blicket kiki fep zup gazzer -> ?
gazzer wif kiki blicket -> ?
fep kiki gazzer wif -> ?
gazzer zup gazzer kiki gazzer wif -> ?
gazzer zup fep kiki dax wif -> ?
```

Figure SI-7: Example prompt that provides five additional episodes to GPT-4 from the MLC meta-learning training set. Note only one of the five episodes is shown here. Problem 6 is the test problem. The tags “> USER:” and “> ASSISTANT:” indicate the roles assigned in the API. These tags did not appear as text in the prompt.

References

[1] Markman, E. M. & Wachtel, G. F. Children's Use of Mutual Exclusivity to Constrain the Meanings of Words. *Cognitive Psychology* **20**, 121–157 (1988).

[2] Gandhi, K. & Lake, B. M. Mutual exclusivity as a challenge for deep neural networks. In *Advances in Neural Information Processing Systems (NeurIPS) 33*, 14182–14192 (2020).

[3] Radford, A. *et al.* Language Models are Unsupervised Multitask Learners. *OpenAI Blog* (2019).

[4] Brown, T. B. *et al.* Language Models are Few-Shot Learners. In *Advances in Neural Information Processing Systems 33 (NeurIPS)* (2020).

[5] OpenAI. GPT-4 Technical Report. *arXiv preprint* (2023). URL `http://arxiv.org/abs/2303.08774`.
