# Supplementary material for: Human-like systematic generalization through a meta-learning neural network
Source: Nature. 2023 Oct 25;623(7985):115–21. doi: 10.1038/s41586-023-06668-3 (PMC10620072; doi:10.1038/s41586-023-06668-3)
Supplement: Supplementary file 2 — Reporting Summary [file 41586_2023_6668_MOESM2_ESM.pdf]

Corresponding author(s): Brenden M. Lake

Last updated by author(s): 09/09/2023

Reporting Summary

Nature Portfolio wishes to improve the reproducibility of the work that we publish. This form provides structure for consistency and transparency in reporting. For further information on Nature Portfolio policies, see our Editorial Policies and the Editorial Policy Checklist.

Statistics

For all statistical analyses, confirm that the following items are present in the figure legend, table legend, main text, or Methods section.

n/a	Confirmed	
☐	☒	The exact sample size (*n*) for each experimental group/condition, given as a discrete number and unit of measurement
☐	☒	A statement on whether measurements were taken from distinct samples or whether the same sample was measured repeatedly
☐	☒	The statistical test(s) used AND whether they are one- or two-sided<br>*Only common tests should be described solely by name; describe more complex techniques in the Methods section.*
☒	☐	A description of all covariates tested
☐	☒	A description of any assumptions or corrections, such as tests of normality and adjustment for multiple comparisons
☐	☒	A full description of the statistical parameters including central tendency (e.g. means) or other basic estimates (e.g. regression coefficient) AND variation (e.g. standard deviation) or associated estimates of uncertainty (e.g. confidence intervals)
☐	☒	For null hypothesis testing, the test statistic (e.g. *F*, *t*, *r*) with confidence intervals, effect sizes, degrees of freedom and *P* value noted<br>*Give P values as exact values whenever suitable.*
☒	☐	For Bayesian analysis, information on the choice of priors and Markov chain Monte Carlo settings
☒	☐	For hierarchical and complex designs, identification of the appropriate level for tests and full reporting of outcomes
☒	☐	Estimates of effect sizes (e.g. Cohen's *d*, Pearson's *r*), indicating how they were calculated

Our web collection on statistics for biologists contains articles on many of the points above.

Software and code

Policy information about availability of computer code

Data collection	Human behavioral data was collected via the psiTurk software (version 2.2.3) and custom code. Code for training and evaluating the MLC models on human behavior is here (https://doi.org/10.5281/zenodo.8274609) and for training and evaluating on machine learning benchmarks is here (https://doi.org/10.5281/zenodo.8274617). For the OpenAI models, GPT-4 was accessed via the Chat Completion API and GPT-3.4 via the Completion API (version text-davinci-003). Any additional custom code is available upon request.
Data analysis	Human behavioral data and analysis code is available here (https://doi.org/10.5281/zenodo.8274609).

For manuscripts utilizing custom algorithms or software that are central to the research but not yet described in published literature, software must be made available to editors and reviewers. We strongly encourage code deposition in a community repository (e.g. GitHub). See the Nature Portfolio guidelines for submitting code & software for further information.

Data

Policy information about availability of data

All manuscripts must include a data availability statement. This statement should provide the following information, where applicable:

- Accession codes, unique identifiers, or web links for publicly available datasets
- A description of any restrictions on data availability
- For clinical datasets or third party data, please ensure that the statement adheres to our policy

Human behavioral data is available here (https://doi.org/10.5281/zenodo.8274609).

Research involving human participants, their data, or biological material

Policy information about studies with human participants or human data. See also policy information about sex, gender (identity/presentation), and sexual orientation and race, ethnicity and racism.

Reporting on sex and gender	This information was not collected.
Reporting on race, ethnicity, or other socially relevant groupings	This information was not collected.
Population characteristics	See study design below
Recruitment	Participants were recruited via Amazon's Mechanical Turk using participants in the United States. These participants may be more comfortable with computers and online surveys than the general population.
Ethics oversight	New York University IRB, FY2018-1728

Note that full information on the approval of the study protocol must also be provided in the manuscript.

Field-specific reporting

Please select the one below that is the best fit for your research. If you are not sure, read the appropriate sections before making your selection.

☐ Life sciences ☒ Behavioural & social sciences ☐ Ecological, evolutionary & environmental sciences

For a reference copy of the document with all sections, see nature.com/documents/nr-reporting-summary-flat.pdf

Behavioural & social sciences study design

All studies must disclose on these points even when the disclosure is negative.

Study description	Quantitative experimental study
Research sample	Participants in the United States were recruited via Amazon's Mechanical Turk. Demographic information was not collected. These participants may be more comfortable with computers and online surveys than the general population, but are likely more representative of the US population than college undergraduates.
Sampling strategy	Random sample. Thirty participants were recruited for each of the two studies in the main text. Twenty-eight participants were recruited for the study in the supporting information. These sample sizes allowed us to observe a number of correct responses and errors for each trial type, and decisively compare models on the fit to human behavior (the best model outperforms the closest alternative by >8 natural log points). The design did not rely on hypothesis testing and thus no power analysis was conducted.
Data collection	Data collection was automated through Amazon's Mechanical Turk and psiTurk.
Timing	Few-shot instruction learning task: 08/02/2018<br>Additional nuances in inductive biases: 08/10/2018<br>Open-ended instruction task: 08/14/2018
Data exclusions	Across the three experiments, there were predesigned catch trials and study quizzes to exclude inattentive participants. In the few-shot instruction learning task, there was one catch trial per stage (except the last stage had two); a participant was excluded if they missed two or more catch trials (N=5). Additionally, each study phase was followed by a quiz, which participants had three chances to get right. Query responses were also excluded if the corresponding study phases were not completed correctly (for all items) within three attempts (13% of remaining data). In the open-ended task, one participant was excluded because they reported using an external aid in a post-test survey. In additional experiment in the supplemental information, two catch trials used query instructions that were identical to a study instruction. Missing a catch trial was the only criterion for exclusion (N = 6).
Non-participation	Few-shot instruction learning task: N=2 opened the experiment but did not begin the task. N=4 began but did not complete the task.<br>Additional nuances in inductive biases: N=2 opened the experiment but did not begin the task. N=4 began but did not complete the task.<br>Open-ended instruction task: N=5 opened the experiment but did not begin the task. N=6 began but did not complete the task.
Randomization	Participants were not allocated to random groups.

Reporting for specific materials, systems and methods

We require information from authors about some types of materials, experimental systems and methods used in many studies. Here, indicate whether each material, system or method listed is relevant to your study. If you are not sure if a list item applies to your research, read the appropriate section before selecting a response.

Materials & experimental systems

n/a	Involved in the study
☒	☐ Antibodies
☒	☐ Eukaryotic cell lines
☒	☐ Palaeontology and archaeology
☒	☐ Animals and other organisms
☒	☐ Clinical data
☒	☐ Dual use research of concern
☒	☐ Plants

Methods

n/a	Involved in the study
☒	☐ ChIP-seq
☒	☐ Flow cytometry
☒	☐ MRI-based neuroimaging
